# Interface of the Polarizable Continuum Model of Solvation with Semi-Empirical Methods in the GAMESS Program

**DOI:** 10.1371/journal.pone.0067725

**Published:** 2013-07-02

**Authors:** Casper Steinmann, Kristoffer L. Blædel, Anders S. Christensen, Jan H. Jensen

**Affiliations:** 1 Department of Physics, Chemistry and Pharmacy, University of Southern Denmark, Odense, Denmark; 2 Department of Chemistry, University of Copenhagen, Copenhagen, Denmark; Jr, Wake Forest University, United States of America

## Abstract

An interface between semi-empirical methods and the polarized continuum model (PCM) of solvation successfully implemented into GAMESS following the approach by Chudinov *et al* (Chem. Phys. 1992, 160, 41). The interface includes energy gradients and is parallelized. For large molecules such as ubiquitin a reasonable speedup (up to a factor of six) is observed for up to 16 cores. The SCF convergence is greatly improved by PCM for proteins compared to the gas phase.

## Introduction

Continuum solvation models such as the polarized continuum model (PCM) [1] and the conductor-like screening model (COSMO) [2] offers a computational efficient model of solvation for molecules treated with electronic structure methods. This paper describes the implementation of an interface between the conductor-PCM (C-PCM) model [2], [3], [4] and the NDDO-based semi-empirical methods implemented in GAMESS [5] (MNDO [6], AM1 [7], and PM3 [8]). There has been several different implementations of semi-empirical/PCM interfaces [2], [9], [10], [11], [12] and this work follows the implementation proposed by Chudinov *et al*. [9] However, we also implement the corresponding energy-gradient terms and both the energy and gradient terms are parallelized and tested on relatively large systems such as the protein ubiquitin.

This paper is organized as follows. 1) We review the relevant expressions for the semi-empirical/PCM interface. 2) We present results of solvation free energies and compare them to previous results. 3) We test the numerical stability for geometry optimizations and vibrational analyses. 4) We present timings and parallelization speed-ups for protein-sized systems. 5) We summarize our findings and provide possible ideas for future improvements.

## Background and Theory

In PCM, a molecule (the solute) is placed inside a solvent-cavity usually described by introducing interlocked spheres placed on the atoms of the molecule. The solvent is described as a polarizable continuum with dielectric constant 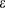. The interaction between the solute and the solvent is described by the apparent surface charges (ASCs). The PCM equations are solved numerically by dividing the surface area up into a finite set of elements called tesserae with a corresponding ASC 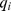, an area 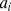 and a position 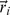. There are several implementations of the PCM [13] and in this study we focus on the conductor-like PCM (C-PCM) [2], [3], [4]. For high dielectric solvents such as water C-PCM yields nearly identical results to the more generally applicable integral-equation-formalism PCM (IEF-PCM) [14] but requires less computational resources.

For C-PCM the ASCs 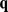 are determined by solving the following matrix-equation
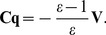
(1)where the matrix 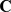 has the elements
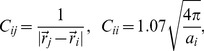
(2)and 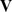 is the potential of the solute in the solvent for each tessera 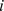. The potential 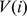 on tessera 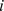 is given as
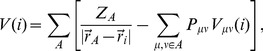
(3)where 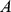 runs over all nuclei in the solute at position 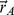 carrying a charge 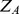. 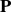 is the density matrix of the solute and 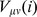 are the interaction integrals over basis functions on a tessera 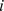 given as



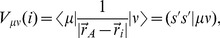
(4)For NDDO methods the right hand side of equation 4 is the interaction between a point charge on the surface (represented as 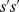 in the NDDO approach) and the basis functions of the solute molecule on atom 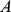. The 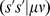 integrals needed in equation 4 are listed in Table 1 for 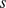 and 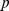 functions. The integrals are rotated from a local ideal coordinate system onto the molecular coordinate system. The local coordinate system is defined by the distance between the atom 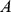 containing the basis functions 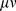 and the tessera 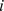


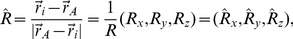
(5)

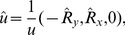
(6)

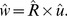
(7)and the four unique integrals from Table 1 are [15]

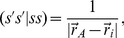
(8)

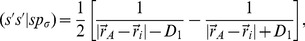
(9)

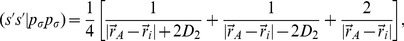
(10)

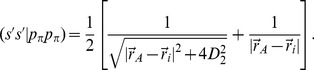
(11)


**Table 1:** Ideal integrals rotated onto the molecular frame.

	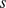	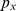	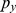	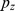
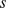	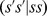	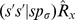	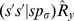	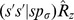
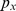		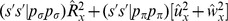	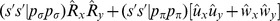	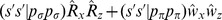
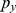			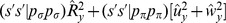	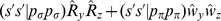
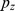				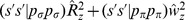

Here, 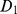 and 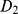 are empirical parameters describing charge-separation for the multipoles. They are defined elsewhere. [15] Following Chudinov *et al.* [9] the density parameters 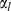 are set to zero in this work and are therefore not shown in the equations.

The electrostatic interaction of the ASCs 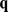 on the surface and the molecule is treated by introducing the following one-electron contribution to the Fock matrix
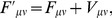
(12)where



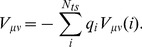
(13)Finally, the PCM electrostatic interaction free energy is calculated as
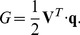
(14)


Optimization of the molecular geometry in the PCM field requires the derivative of 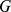 with respect to an atomic coordinate 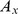


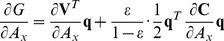
(15)the last term is computed analytically [16]. The derivative of the potential with respect to an atomic coordinate is done analytically and we give explicit expressions for all terms in Text S1.

## Methods

### Computational Details

The semi-empirical/PCM interface was implemented in a locally modified version of GAMESS [5]. The semi-empirical energy and gradient evaluations were allowed to run in parallel but no efforts were made to parallelize the integral evaluation or the assembly of the Fock matrix since the diagonalization is the major computational bottle-neck for large systems. The evaluation of the electrostatic potential (equation 3) and its derivative (equation 15) was parallelized. We note that the remaining semi-empirical integral-derivatives in GAMESS is evaluated numerically.

We compared our implementation to that of Chudinov *et al.* for twenty smaller ammonium and oxonium type molecules used in that study. The structures were generated from their SMILES string (see Table 2 and Table 3) using Open Babel [17], [18] and optimized in the gas phase and afterwards using the newly implemented code.

**Table 2:** Predicted electrostatic solvation free energies of ammonium type molecules.

		Ref	PM3/PCM		RHF/STO-3G/PCM	
[NH4+]	A1	83.9	82.4	(−1.5)	78.6	(−3.8)
C[NH3+]	A2	73.7	72.6	(−1.1)	71.3	(−1.3)
CC[NH3+]	A3	70.2	69.2	(−1.0)	68.6	(−0.6)
CCC[NH3+]	A4	69.9	68.5	(−0.8)	67.6	(−1.0)
CC([NH3+])C	A5	67.1	65.9	(−1.2)	66.2	(0.3)
CCCC[NH3+]	A6	69.3	68.3	(−1.0)	67.1	(−1.2)
CC([NH3+])(C)C	A7	64.1	62.8	(−1.3)	67.1	(1.2)
C[NH2+]C	A8	65.9	64.4	(−1.5)	65.3	(0.9)
CC[NH2+]CC	A9	59.5	58.0	(−1.5)	60.7	(2.7)
C[NH+](C)C	A10	59.7	57.7	(−2.1)	61.8	(4.2)
AVG			−1.3			

Obtained results using PM3/PCM compared with results by Chudinov *et al*. (labelled "Ref") and RHF/STO-3G/PCM results. PM3/PCM numbers in parenthesis are deviations to the reference. RHF/STO-3G deviations are taken to PM3/PCM results. All numbers are in kcal mol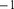.

**Table 3:** Predicted electrostatic solvation free energies of oxonium type molecules.

		Ref	PM3/PCM		RHF/STO-3G/PCM	
C[OH2+]	O1	74.1	72.6	(−1.5)	73.7	(3.0)
CC[OH2+]	O2	69.2	67.1	(−2.1)	70.2	(3.0)
C[OH+]C	O3	65.1	63.4	(−1.7)	65.5	(2.1)
C[OH+]CC	O4	61.1	59.0	(−2.1)	62.5	(3.5)
C1C[OH+]CC1	O5	59.6	57.3	(−2.3)	61.0	(3.8)
CC[OH+]CC	O6	57.4	55.4	(−2.0)	59.8	(4.1)
C[OH+]c1ccccc1	O7	54.5	53.3	(−1.2)	57.4	(4.4)
CC( = [OH+])C	O8	62.5	60.0	(−2.5)	64.3	(4.3)
CC(C)C( = [OH+])C(C)C	O9	53.2	51.0	(−2.2)	56.0	(5.0)
COC( = [OH+])C	O10	60.0	58.7	(−1.3)	62.6	(3.9)
AVG			−2.0			

Obtained results using PM3/PCM compared with results by Chudinov *et al*. (labelled "Ref") and RHF/STO-3G/PCM results. PM3/PCM numbers in parenthesis are deviations to the reference. RHF/STO-3G deviations are taken to PM3/PCM results. All numbers are in kcal mol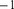.

Geometry optimizations used a convergence threshold of 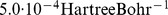 (OPTTOL = 5.0E-4 in $STATPT). To verify the minima, hessians were calculated for all optimized geometries by double difference (NVIB = 2 in $FORCE). When using PCM for geometry optimizations the FIXPVA [19] tessellation scheme was used (MTHALL = 4 in $TESCAV) and the tesserae count for each sphere was set to 60 (NTSALL = 60 in $TESCAV). For solvation free energies the tesserae count was raised to 960 (NTSALL = 960 in $TESCAV) and the GEPOL-GB (Gauss-Bonet) [20] tessellation scheme (MTHALL = 1 in $TESCAV) was used.

The Mean Absolute Deviations (MADs) of vibrational frequencies between solvated (s) and gas-phase (g) calculations were calculated by
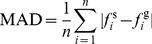
(16)


We also carried out single point energies and gradients calculations for Chignolin (PDB: 1UAO), Trypthophan-cage (PDB: 1L2Y), Crambine (PDB: 1CRN), Trypsin Inhibitor (PDB: 5PTI) and Ubiquitin (PDB: 1UBI). The proteins were all protonated with PDB2PQR [21], [22] and PROPKA [23] at 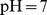 yielding overall charges of −2, 1, 0, 6 and 0 respectively. Either no convergence acceleration, Direct Inversion of the Iterative Subspace [24] (DIIS = .T. in $SCF) or Second-Order Self Consistent Field [25], [26] (SOSCF = .T. in $SCF) was used. In all cases the C-PCM equation was solved iteratively. [27] The timings were performed on up to 24 cores on AMD Optirun 6172 shared-memory CPUs. The method is included in the latest release of the GAMESS program.

## Results and Discussion

### Electrostatic Solvation Free Energies

The electrostatic solvation free energies are presented in Tables 2 and 3 for ammonium and oxonium species calculated using PM3/PCM and compared to results published by Chudinov *et al*. [9] In general, our results underestimate the electrostatic solvation free energy by an average of −1.3 kcal mol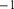 and −1.9 kcal mol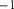. The main source of the difference is likely the fact that Chudinov *et al.* uses the original PCM implementation of Miertus, Scrocco and Tomasi [28] often referred to a D-PCM) while we use the C-PCM implementation. The solvation free energies from these implementations can differ by several kcal/mol even for neutral molecules [29]. (While the reference describes a comparison of D-PCM to IEF-PCM, IEF-PCM and C-PCM yield nearly identical solvation free energies for water.) Another likely source of error is that we use the GEPOL-GB scheme where Chudinov *et al.* uses a more elaborate scheme to reach convergence of the solvation free energies by subdividing the surfaces incrementally.

### Vibrational Frequencies

To test the numerical accuracy of the PCM gradients we optimized the molecules listed in Tables 2 and 3. As indicated in Table 4 three of the geometry optimizations (A1, O1, and O2) do not converge. A1 can be made to converge by skipping the update of the empirical Hessian matrix (UPHESS = SKIP) but this does not appear to be a general solution to the problem. While some gradient components in these minimizations are quite large the optimizing algorithm eventually settles on a zero step size causing the optimization to effectively stall. The cause of this behavior is not clear since it is only observed for the smallest systems and was not investigated further. The resulting geometries still lead to a positive definite Hessian and the frequencies are not unusually different from the gas phase values.

**Table 4:** Optimization steps and frequencies for solvated molecules.

	*N*_steps_		MAD [cm 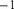 ]	
	PM3	RHF/STO-3G	PM3	RHF/STO-3G
A1	–	14	135.1	131.9
A2	9	10	121.5	90.8
A3	6	8	64.6	39.2
A4	6	18	25.7	37.9
A5	4	17	16.9	24.6
A6	10	9	30.4	15.5
A7	32	32	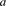 24.8	22.3
A8	25	24	56.2	32.8
A9	34	19	27.3	31.3
A10	32	18	58.3	62.2
O1	–	6	151.8	60.1
O2	–	8	111.5	36.2
O3	15	8	96.8	57.0
O4	6	8	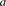 67.1	28.3
O5	11	9	85.6	29.8
O6	15	11	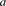 56.0	54.5
O7	6	6	50.1	24.5
O8	11	7	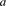 87.7	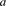 22.0
O9	6	8	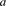 28.8	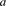 12.6
O10	3	6	20.8	19.9

Number of optimization steps for PM3/PCM and RHF/STO-3G/PCM optimizations along with Mean Absolute Deviations (MADs) of vibrational frequencies when going from gas phase to a solvated molecule for all 20 small molecules tested in this work. All optimizations were done in Cartesian coordinates. Translational and rotational frequencies are not included. Dashes marks unconverged structures after 100 optimization steps.

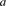
marks optimized structures with at least one imaginary frequency.

In four cases (A7, O4, O6, O8 and O9) the vibrational analyses yields imaginary frequencies between 26 and 200 cm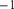. In the case of O8 and O9 this also occurs for the RHF/STO-3G calculations and in the case of O7–O9 this also occurs for PM3 structures optimized in the gas phase. In most cases the imaginary frequency is associated with the O+ ion and a neighbouring methyl group. The most likely source of these imaginary frequencies is a flat PES associated with the O+ group combined with numerical inaccuracies in the PCM and PM3 gradients.

### Timings

In Table 5 we show absolute timings for single point energy and gradient evaluations of proteins either in the gas phase, using DIIS to obtain convergence, or by including the PCM field either with or without SCF convergence acceleration. None of the listed proteins converged in the gas phase without DIIS and even then the SCF converged only for the three smallest proteins: Chignolin, Tryptophan-Cage and Crambine.

**Table 5:** Absolute timings for energy and gradient evaluations.

System	PDB	*N_at_*	*N_tes_*	DIIS	PCM	PCM/DIIS	PCM/SOSCF	
Chignolin	1UAO	138	1355	6	29	21	21	(0.4)
Trp-cage	1L2Y	304	2609	61	159	158	141	(1.4)
Crambine	1CRN	642	4112	563	1293	1314	1277	(6)
Trypsin Inhibitor	5PTI	892	6315	–	3455	3649	3409	(12)
Ubiquitin	1UBI	1231	7956	–	6732	8777	7607	(22)

Absolute timings in seconds for energy and gradient evaluation for various proteins using both gas phase PM3 and PM3/PCM. Numbers in parenthesis are analytic electronic field gradient timings used in the analytical PM3/PCM gradient. No gas phase SCF converged without DIIS.

The cost of optimizing the wavefunction in PCM is between two (Crambine) and three (Chignolin and Tryptophan-cage) times more expensive than without. For Chignolin, which is the smallest protein in our test set, it took 21 SCF iterations to converge in PCM while only 13 for PCM/DIIS and 14 for PCM/SOSCF. The other proteins converged within 17 iterations without convergence acceleration and within 14 iterations with. For absolute timings regarding larger proteins, Crambine, Trypsin Inhibitor and Ubiquitin finished in 1293, 3455 and 6732 seconds with PCM without convergence accelleration, but are slower (1314, 3649 and 8777 seconds, respectively) with PCM and DIIS enabled. Using SOSCF did not result in an appreciable decrease in CPU time. The increase in CPU time when using DIIS is due to the extra matrix operations associated with this method, which represent the computational bottleneck for sem-empirical methods.

Evaluating the ASC potential derivative (equation 15) analytically has a negligible computational cost compared to evaluating the wavefunction as can be seen from the last column of Table 5.

**Figure 1:**
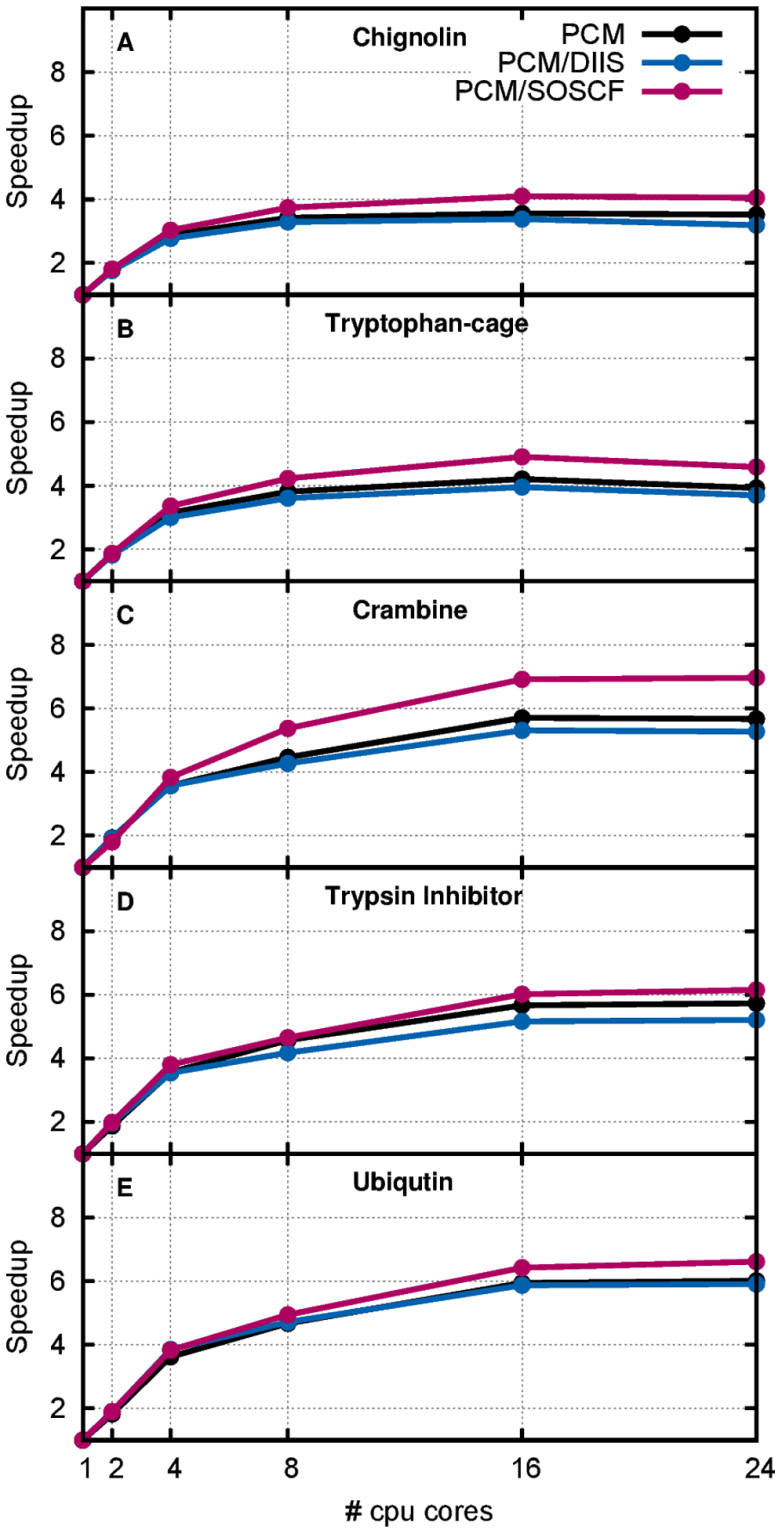
Speedup by using multiple cores with PCM enabled for single gradient evaluation.

The relative speedup from running in parallel in the gas phase is shown on Figure S1 where no improvement is observed beyond 4 cores (with a speed up factor of 3) and is not discussed further. The PM3/PCM timings (Figure 1) show better improvement when utilizing multiple cores for all systems. The smaller systems obtain some improvement (a factor 3.4 and 4.2 for Chignolin and Tryptophan-cage, respectively) whereas the larger systems sees improvements of 5.7, 5.7 and 5.9 for Crambine, Trypsin Inhibitor and Ubiquitin, respectively. In all cases maximum speed up is reached for 16 cores because the use of 24 cores introduces some communication overhead which degraded performance.

### Conclusions

An interface between semi-empirical methods and the polarized continuum model (PCM) of solvation successfully implemented into GAMESS following the approach by Chudinov *et al*. [9] The interface includes energy gradients and is parallelized.

For very small systems we found some numerical instability problems in the gradient which caused geometry convergence failure, but geometry optimization appears robust for larger molecules. The use of PCM occasionally introduces imaginary frequencies in the Hessian analysis, but this was also found for RHF/STO-3G PCM calculations and even in a few semi-empirical gas phase calculations so these problems do not appear to be specific to the to the current implementation. We therefore consider the current implementation a working code for all practical purposes, but welcome feedback from readers who encounter numerical stability problems for large molecules.

For semiemprical methods the most time CPU-intensive part of the calculation remains the solution of the SCF equations. This part of the code was already parallelized in GAMESS and we show, for the first time, that this implementation applies to semi-empirical methods and the new PCM interface. For large molecules such as Ubiquitin a reasonable speedup (up to a factor of six) is observed for up to 16 cores.

It will be interesting to see how much the numerical stability and computational efficiency will improve once the interface is combined with the recently developed FIXSOL/FIXPVA2 method developed by Li and coworkers [30]. We are currently working on implementing the PM6 method in GAMESS to further increase the accuracy and range of application that this new interface offers.

## Supporting Information

Text S1 **Analytical derivative of the interaction potential.**
(PDF)

Figure S1 **Speedup by using multiple cores single gas phase gradient evaluation.**
(TIFF)

## References

[1] Mennucci B (2012) Polarizable continuum model. Wiley Interdisciplinary Reviews: Computational Molecular Science 2: 386–404.

[2] Klamt A, Schüürmann G (1993) Cosmo: a new approach to dielectric screening in solvents with explicit expressions for the screening energy and its gradient. J Chem Soc : 799–805.

[3] Barone V, Cossi M (1998) Quantum calculation of molecular energies and energy gradients in solution by a conductor solvent model. J Phys Chem A 102: 1995– 2001.

[4] Cossi M, Rega N, Scalmani G, Barone V (2003) Energies, structures, and electronic properties of molecules in solution with the c-pcm solvation model. J Comput Chem 24: 669–681. 12666158 10.1002/jcc.10189

[5] Schmidt MW, Baldridge KK, Boatz JA, Elbert S, Gordon MS, et al (1993) General atomic molecular electronic structure system. J Comput Chem 14: 1347–1363.

[6] Dewar MJS, Thiel W (1977) Ground states of molecules. 38. the mndo method. approximations and parameters. J Am Chem Soc 99: 4899–4907.

[7] Dewar MJS, Zoebisch EG, Healy EF, Stewart JJP (1985) Development and use of quantum mechanical molecular models. 76. am1: a new general purpose quantum mechanical molecular model. J Am Chem Soc 107: 3902–3909.

[8] Stewart JJP (1989) Optimization of parameters for semiempirical methods i. method. J Comp Chem 10: 209–220.

[9] Chudinov GE, Napolov DV, Basilevsky MV (1992) Quantum-chemical calculations of the hydration energies of organic cations and anions in the framework of a continuum solvent approximation. Chem Phys 160: 41–54.

[10] Negre MJ, Orozco M, Luque FJ (1992) A new strategy for the representation of environment effects in semi-empirical calculations based on dewar's hamiltonians. Chem Phys Lett 196: 27–36.

[11] Luque FJ, Negre MJ, Orozco M (1993) An am1-scrf approach to the study of changes in molecular properties induced by solvent. J Phys Chem 97: 4386–4391.

[12] Caricato M, Mennucci B, Tomasi J (2004) Solvent effects on the electronic spectra: An extension of the polarizable continuum model to the zindo method. J Phys Chem A 108: 6248–6256.

[13] Tomasi J, Mennucci B, Cammi R, et al (2005) Quantum mechanical continuum solvation models. Chem Rev 105: 2999–3094. 16092826 10.1021/cr9904009

[14] Tomasi J, Mennucci B, Cances E (1999) The ief version of the pcm salvation method: an overview of a new method addressed to study molecular solutes at the qm ab initio level. J Mol Struct THEOCHEM 464: 211–226.

[15] Dewar MJS, Thiel W (1977) A semiempirical model for the two-center repulsion integrals in the nddo approximation. Theor Chem Acc 46: 89–104.

[16] Li H, Jensen JH (2004) Improving the effciency and convergence of geometry optimization with the polarizable continuum model: New energy gradients and molecular surface tessellation. J Comp Chem 25: 1449–1462. 15224389 10.1002/jcc.20072

[17] Openbabel v.2.3.2. URL http://openbabel.sourceforge.net. Accessed 2013 Jan 10.

[18] O'Boyle NM, Banck M, James CA, Morley C, Vandermeersch T, et al (2011) Open babel: An open chemical toolbox. J Cheminf 3: 33. 10.1186/1758-2946-3-33 PMC3198950 21982300

[19] Su P, Li H (2009) Continuous and smooth potential energy surface for conductorlike screening solvation model using fixed points with variable areas. J Chem Phys 130: 074109. 19239286 10.1063/1.3077917

[20] Pascual-Ahuir J, Silla E, Tomasi J, Bonaccorsi R (1987) Electrostatic interaction of a solute with a continuum. improved description of the cavity and of the surface cavity bound charge distribution. J Comput Chem 8: 778–787.

[21] Dolinsky TJ, Nielsen JE, McCammon JA, Baker NA (2004) PDB2PQR: an automated pipeline for the setup of Poisson–Boltzmann electrostatics calculations. Nucleic Acids Res 32: W665. 15215472 10.1093/nar/gkh381 PMC441519

[22] Dolinsky TJ, Czodrowski P, Li H, Nielsen JE, Jensen JH, et al (2007) PDB2PQR: expanding and upgrading automated preparation of biomolecular structures for molecular simulations. Nucleic Acids Res 35: W522. 17488841 10.1093/nar/gkm276 PMC1933214

[23] Li H, Robertson AD, Jensen JH (2005) Very fast empirical prediction and rationalization of protein pka values. Proteins 61: 704–721. 16231289 10.1002/prot.20660

[24] Pulay P (1982) Improved scf convergence acceleration. J Comput Chem 3: 556– 560.

[25] Fischer TH, Almlof J (1992) General methods for geometry and wave function optimization. J Phys Chem 96: 9768–9774.

[26] Chaban G, Schmidt MW, Gordon MS (1997) Approximate second order method for orbital optimization of scf and mcscf wavefunctions. Theor Chem Acc 97: 88–95.

[27] Li H, Pomelli CS, Jensen JH (2003) Continuum solvation of large molecules described by qm/mm: a semi-iterative implementation of the pcm/efp interface. Theor Chem Acc 109: 71–84.

[28] Miertuš S, Scrocco E, Tomasi J (1981) Electrostatic interaction of a solute with a continuum. a direct utilizaion of ab initio molecular potentials for the prevision of solvent effects. Chem Phys 55: 117–129.

[29] Cances E, Mennucci B, Tomasi J (1997) A new integral equation formalism for the polarizable continuum model: Theoretical background and applications to isotropic and anisotropic dielectrics. J Chem Phys 107: 3032–3041.

[30] Thellamurege NM, Li H (2012) Note: Fixsol solvation model and fixpva2 tessellation scheme. J Chem Phys 137: 246101. 23277961 10.1063/1.4773280

