# Supplementary material for: Interface of the Polarizable Continuum Model of Solvation with Semi-Empirical Methods in the GAMESS Program
Source: PLoS One. 2013 Jul 2;8(7):e67725. doi: 10.1371/journal.pone.0067725 (PMC3699639; doi:10.1371/journal.pone.0067725)
Supplement: Text S1 — Analytical derivative of the interaction potential. (PDF) [file pone.0067725.s001.pdf]

restart;
#Digits:=40;
with(LinearAlgebra) :

Text S1

This document evaluates the derived potential dV/dAx

Ideal integrals

$$SSSS := (Rx, Ry, Rz, Tx, Ty, Tz) \rightarrow \frac{1}{\mathrm{sqrt}\left((Rx-Tx)^2+(Ry-Ty)^2+(Rz-Tz)^2\right)};$$

$$(Rx, Ry, Rz, Tx, Ty, Tz) \rightarrow \frac{1}{\sqrt{(Rx-Tx)^2+(Ry-Ty)^2+(Rz-Tz)^2}} \quad \textbf{(1.1)}$$

$$SSPOPO := (Rx, Ry, Rz, Tx, Ty, Tz, D2) \rightarrow SSSS(Rx, Ry, Rz, Tx, Ty, Tz) + \frac{1}{2}\left(\frac{1}{\mathrm{sqrt}\left((Rx-Tx)^2+(Ry-Ty)^2+(Rz-Tz)^2+(2\cdot D2)^2\right)} - SSSS(Rx, Ry, Rz, Tx, Ty, Tz)\right);$$

$$(Rx, Ry, Rz, Tx, Ty, Tz, D2) \rightarrow \frac{1}{2}\,SSSS(Rx, Ry, Rz, Tx, Ty, Tz) + \frac{1}{2\sqrt{(Rx-Tx)^2+(Ry-Ty)^2+(Rz-Tz)^2+4\,D2^2}} \quad \textbf{(1.2)}$$

$$SSPZPZ := (Rx, Ry, Rz, Tx, Ty, Tz, D2) \rightarrow SSSS(Rx, Ry, Rz, Tx, Ty, Tz) + \frac{1}{4}\left(\frac{1}{\mathrm{sqrt}\left((Rx-Tx)^2+(Ry-Ty)^2+(Rz-Tz)^2\right)+2\,D2} + \frac{1}{\mathrm{sqrt}\left((Rx-Tx)^2+(Ry-Ty)^2+(Rz-Tz)^2\right)-2\,D2}\right) - \frac{1}{2}\cdot SSSS(Rx, Ry, Rz, Tx, Ty, Tz);$$

$$(Rx, Ry, Rz, Tx, Ty, Tz, D2) \rightarrow \frac{1}{2}\,SSSS(Rx, Ry, Rz, Tx, Ty, Tz) + \frac{1}{4\left(\sqrt{(Rx-Tx)^2+(Ry-Ty)^2+(Rz-Tz)^2}+2\,D2\right)} + \frac{1}{4\left(\sqrt{(Rx-Tx)^2+(Ry-Ty)^2+(Rz-Tz)^2}-2\,D2\right)} \quad \textbf{(1.3)}$$

$$SSSPZ := (Rx, Ry, Rz, Tx, Ty, Tz, D1) \rightarrow \frac{1}{2}\cdot\left(\frac{1}{\mathrm{sqrt}\left((Rx-Tx)^2+(Ry-Ty)^2+(Rz-Tz)^2\right)+\mathrm{D1}} - \frac{1}{\mathrm{sqrt}\left((Rx-Tx)^2+(Ry-Ty)^2+(Rz-Tz)^2\right)-D1}\right);$$

$$(Rx, Ry, Rz, Tx, Ty, Tz, D1) \rightarrow \frac{1}{2\left(\sqrt{(Rx-Tx)^2+(Ry-Ty)^2+(Rz-Tz)^2}+D1\right)} \quad \textbf{(1.4)}$$

$$-\frac{1}{2\left(\sqrt{(Rx-Tx)^2+(Ry-Ty)^2+(Rz-Tz)^2}-D1\right)}$$

Projection operators

$$X_1 := (Rx, Ry, Rz, Tx, Ty, Tz) \rightarrow \frac{Rx-Tx}{\mathrm{sqrt}\left((Rx-Tx)^2+(Ry-Ty)^2+(Rz-Tz)^2\right)} :$$

$X_1(Rx, Ry, Rz, Tx, Ty, Tz)$:

$subs\left(Rx^2-2\,Rx\,Tx+Tx^2+Ry^2-2\,Ry\,Ty+Ty^2+Rz^2-2\,Rz\,Tz+Tz^2=R2, \%\right)$:

$factor(\%)$;

$$\frac{Rx-Tx}{\sqrt{R2}} \quad \textbf{(2.1)}$$

$$X_2 := (Rx, Ry, Rz, Tx, Ty, Tz) \rightarrow \frac{Ry-Ty}{\mathrm{sqrt}\left((Rx-Tx)^2+(Ry-Ty)^2+(Rz-Tz)^2\right)} :$$

$X_2(Rx, Ry, Rz, Tx, Ty, Tz)$:

$subs\left(Rx^2-2\,Rx\,Tx+Tx^2+Ry^2-2\,Ry\,Ty+Ty^2+Rz^2-2\,Rz\,Tz+Tz^2=R2, \%\right)$:

$factor(\%)$;

$$\frac{Ry-Ty}{\sqrt{R2}} \quad \textbf{(2.2)}$$

$$X_3 := (Rx, Ry, Rz, Tx, Ty, Tz) \rightarrow \frac{Rz-Tz}{\mathrm{sqrt}\left((Rx-Tx)^2+(Ry-Ty)^2+(Rz-Tz)^2\right)} :$$

$X_3(Rx, Ry, Rz, Tx, Ty, Tz)$:

$subs\left(Rx^2-2\,Rx\,Tx+Tx^2+Ry^2-2\,Ry\,Ty+Ty^2+Rz^2-2\,Rz\,Tz+Tz^2=R2, \%\right)$:

$factor(\%)$;

$$\frac{Rz-Tz}{\sqrt{R2}} \quad \textbf{(2.3)}$$

$$Y_1 := (Rx, Ry, Rz, Tx, Ty, Tz) \rightarrow -\frac{Ry-Ty}{\mathrm{sqrt}\left((Rx-Tx)^2+(Ry-Ty)^2\right)} :$$

$Y_1(Rx, Ry, Rz, Tx, Ty, Tz)$:

$subs\left(Rx^2-2\,Rx\,Tx+Tx^2+Ry^2-2\,Ry\,Ty+Ty^2+Rz^2-2\,Rz\,Tz+Tz^2=R2, \%\right)$:

$subs\left(Rx^2-2\,Rx\,Tx+Tx^2+Ry^2-2\,Ry\,Ty+Ty^2=RY2, \%\right)$:

$factor(\%)$;

$$-\frac{Ry-Ty}{\sqrt{RY2}} \quad \textbf{(2.4)}$$

$$Y_2 := (Rx, Ry, Rz, Tx, Ty, Tz) \rightarrow \frac{Rx-Tx}{\mathrm{sqrt}\left((Rx-Tx)^2+(Ry-Ty)^2\right)} :$$

$Y_2(Rx, Ry, Rz, Tx, Ty, Tz)$:

$subs\left(Rx^2-2\,Rx\,Tx+Tx^2+Ry^2-2\,Ry\,Ty+Ty^2+Rz^2-2\,Rz\,Tz+Tz^2=R2, \%\right)$:

$subs\left(Rx^2-2\,Rx\,Tx+Tx^2+Ry^2-2\,Ry\,Ty+Ty^2=RY2, \%\right)$:

$factor(\%)$;

$$\frac{Rx - Tx}{\sqrt{RY2}} \quad (2.5)$$

$Z_1 := (Rx, Ry, Rz, Tx, Ty, Tz) \rightarrow$

$$-\frac{(Rx - Tx)\cdot(Rz - Tz)}{\mathrm{sqrt}\left((Rx - Tx)^2 + (Ry - Ty)^2 + (Rz - Tz)^2\right)\cdot \mathrm{sqrt}\left((Rx - Tx)^2 + (Ry - Ty)^2\right)} :$$

$Z_1(Rx, Ry, Rz, Tx, Ty, Tz) :$

$subs\left(Rx^2 - 2\,Rx\,Tx + Tx^2 + Ry^2 - 2\,Ry\,Ty + Ty^2 + Rz^2 - 2\,Rz\,Tz + Tz^2 = R2, \%\right) :$

$subs\left(Rx^2 - 2\,Rx\,Tx + Tx^2 + Ry^2 - 2\,Ry\,Ty + Ty^2 = RY2, \%\right) :$

$factor(\%);$

$$-\frac{(Rx - Tx)\;(Rz - Tz)}{\sqrt{R2}\;\sqrt{RY2}} \quad (2.6)$$

$Z_2 := (Rx, Ry, Rz, Tx, Ty, Tz) \rightarrow$

$$-\frac{(Ry - Ty)\cdot(Rz - Tz)}{\mathrm{sqrt}\left((Rx - Tx)^2 + (Ry - Ty)^2 + (Rz - Tz)^2\right)\cdot \mathrm{sqrt}\left((Rx - Tx)^2 + (Ry - Ty)^2\right)} :$$

$Z_2(Rx, Ry, Rz, Tx, Ty, Tz) :$

$subs\left(Rx^2 - 2\,Rx\,Tx + Tx^2 + Ry^2 - 2\,Ry\,Ty + Ty^2 + Rz^2 - 2\,Rz\,Tz + Tz^2 = R2, \%\right) :$

$subs\left(Rx^2 - 2\,Rx\,Tx + Tx^2 + Ry^2 - 2\,Ry\,Ty + Ty^2 = RY2, \%\right) :$

$factor(\%);$

$$-\frac{(Ry - Ty)\;(Rz - Tz)}{\sqrt{R2}\;\sqrt{RY2}} \quad (2.7)$$

$Z_3 := (Rx, Ry, Rz, Tx, Ty, Tz)$

$$\rightarrow \frac{(Rx - Tx)^2 + (Ry - Ty)^2}{\mathrm{sqrt}\left((Rx - Tx)^2 + (Ry - Ty)^2 + (Rz - Tz)^2\right)\cdot \mathrm{sqrt}\left((Rx - Tx)^2 + (Ry - Ty)^2\right)} :$$

$Z_3(Rx, Ry, Rz, Tx, Ty, Tz) :$

$subs\left(Rx^2 - 2\,Rx\,Tx + Tx^2 + Ry^2 - 2\,Ry\,Ty + Ty^2 + Rz^2 - 2\,Rz\,Tz + Tz^2 = R2, \%\right) :$

$subs\left(Rx^2 - 2\,Rx\,Tx + Tx^2 + Ry^2 - 2\,Ry\,Ty + Ty^2 = RY2, \%\right) :$

$factor(\%);$

$$\frac{Rx^2 - 2\,Rx\,Tx + Tx^2 + Ry^2 - 2\,Ry\,Ty + Ty^2}{\sqrt{R2}\;\sqrt{RY2}} \quad (2.8)$$

Derived ideal integrals

Here the simple derivative of the integrals are evaluated.

1) First we have the (ss|ss) integrals

$diff\,(SSSS(Rx, Ry, Rz, Tx, Ty, Tz), Rx) :$

$subs\left(Rx^2 - 2\,Rx\,Tx + Tx^2 + Ry^2 - 2\,Ry\,Ty + Ty^2 + Rz^2 - 2\,Rz\,Tz + Tz^2 = R2, \%\right) :$

$factor(\%);$

$$-\frac{Rx - Tx}{R2^{3/2}} \quad (3.1)$$

$diff\,(SSSS(Rx, Ry, Rz, Tx, Ty, Tz), Ry) :$

$subs\left(Rx^2 - 2\,Rx\,Tx + Tx^2 + Ry^2 - 2\,Ry\,Ty + Ty^2 + Rz^2 - 2\,Rz\,Tz + Tz^2 = R2, \%\right) :$

factor(%);

$$-\frac{Ry - Ty}{R2^{3/2}} \quad \textbf{(3.2)}$$

diff(*SSSS*(*Rx*, *Ry*, *Rz*, *Tx*, *Ty*, *Tz*), *Rz*) :
$subs\left(Rx^2 - 2\,Rx\,Tx + Tx^2 + Ry^2 - 2\,Ry\,Ty + Ty^2 + Rz^2 - 2\,Rz\,Tz + Tz^2 = R2, \%\right)$:
factor(%);

$$-\frac{Rz - Tz}{R2^{3/2}} \quad \textbf{(3.3)}$$

2) Second we have the (ss|p_op_o) integrals
diff(*SSPOPO*(*Rx*, *Ry*, *Rz*, *Tx*, *Ty*, *Tz*, *D2*), *Rx*);

$$-\frac{1}{4}\,\frac{2\,Rx - 2\,Tx}{\left(Rx^2 - 2\,Rx\,Tx + Tx^2 + Ry^2 - 2\,Ry\,Ty + Ty^2 + Rz^2 - 2\,Rz\,Tz + Tz^2\right)^{3/2}} - \frac{1}{4}\,\frac{2\,Rx - 2\,Tx}{\left(Rx^2 - 2\,Rx\,Tx + Tx^2 + Ry^2 - 2\,Ry\,Ty + Ty^2 + Rz^2 - 2\,Rz\,Tz + Tz^2 + 4\,D2^2\right)^{3/2}} \quad \textbf{(3.4)}$$

(3.5)

(3.6)

diff(*SSPOPO*(*Rx*, *Ry*, *Rz*, *Tx*, *Ty*, *Tz*, *D2*), *Ry*);

$$-\frac{1}{4}\,\frac{2\,Ry - 2\,Ty}{\left(Rx^2 - 2\,Rx\,Tx + Tx^2 + Ry^2 - 2\,Ry\,Ty + Ty^2 + Rz^2 - 2\,Rz\,Tz + Tz^2\right)^{3/2}} - \frac{1}{4}\,\frac{2\,Ry - 2\,Ty}{\left(Rx^2 - 2\,Rx\,Tx + Tx^2 + Ry^2 - 2\,Ry\,Ty + Ty^2 + Rz^2 - 2\,Rz\,Tz + Tz^2 + 4\,D2^2\right)^{3/2}} \quad \textbf{(3.7)}$$

(3.8)

(3.9)

diff(*SSPOPO*(*Rx*, *Ry*, *Rz*, *Tx*, *Ty*, *Tz*, *D2*), *Rz*);

$$-\frac{1}{4}\,\frac{2\,Rz - 2\,Tz}{\left(Rx^2 - 2\,Rx\,Tx + Tx^2 + Ry^2 - 2\,Ry\,Ty + Ty^2 + Rz^2 - 2\,Rz\,Tz + Tz^2\right)^{3/2}} - \frac{1}{4}\,\frac{2\,Rz - 2\,Tz}{\left(Rx^2 - 2\,Rx\,Tx + Tx^2 + Ry^2 - 2\,Ry\,Ty + Ty^2 + Rz^2 - 2\,Rz\,Tz + Tz^2 + 4\,D2^2\right)^{3/2}} \quad \textbf{(3.10)}$$

(3.11)

(3.12)

3) Third we have the (ss|p_sp_s) integrals
diff(*SSPZPZ*(*Rx*, *Ry*, *Rz*, *Tx*, *Ty*, *Tz*, *D2*), *Rx*) :
$subs\left(Rx^2 - 2\,Rx\,Tx + Tx^2 + Ry^2 - 2\,Ry\,Ty + Ty^2 + Rz^2 - 2\,Rz\,Tz + Tz^2 = R2, \%\right)$:
factor(%);

$$-\frac{(Rx - Tx)\left(R2^2 - 2\,R2\,D2^2 + 8\,D2^4\right)}{R2^{3/2}\left(\sqrt{R2} + 2\,D2\right)^2\left(-\sqrt{R2} + 2\,D2\right)^2} \quad \textbf{(3.13)}$$

diff(*SSPZPZ*(*Rx*, *Ry*, *Rz*, *Tx*, *Ty*, *Tz*, *D2*), *Ry*) :
$subs\left(Rx^2 - 2\,Rx\,Tx + Tx^2 + Ry^2 - 2\,Ry\,Ty + Ty^2 + Rz^2 - 2\,Rz\,Tz + Tz^2 = R2, \%\right)$:
factor(%);

$$-\frac{(Ry-Ty)\,(R2^2-2\,R2\,D2^2+8\,D2^4)}{R2^{3/2}\,(\sqrt{R2}+2\,D2)^2\,(-\sqrt{R2}+2\,D2)^2} \tag{3.14}$$

$diff(SSPZPZ(Rx, Ry, Rz, Tx, Ty, Tz, D2), Rz)$:
$subs(Rx^2-2\,Rx\,Tx+Tx^2+Ry^2-2\,Ry\,Ty+Ty^2+Rz^2-2\,Rz\,Tz+Tz^2=R2, \%)$:
$factor(\%)$;

$$-\frac{(Rz-Tz)\,(R2^2-2\,R2\,D2^2+8\,D2^4)}{R2^{3/2}\,(\sqrt{R2}+2\,D2)^2\,(-\sqrt{R2}+2\,D2)^2} \tag{3.15}$$

4) Finally we have the (ss|sp_s) integrals
$diff(SSSPZ(Rx, Ry, Rz, Tx, Ty, Tz, D1), Rx)$:
$subs(Rx^2-2\,Rx\,Tx+Tx^2+Ry^2-2\,Ry\,Ty+Ty^2+Rz^2-2\,Rz\,Tz+Tz^2=R2, \%)$:
$factor(\%)$;

$$\frac{2\,(Rx-Tx)\,D1}{(\sqrt{R2}+D1)^2\,(-\sqrt{R2}+D1)^2} \tag{3.16}$$

$diff(SSSPZ(Rx, Ry, Rz, Tx, Ty, Tz, D1), Ry)$:
$subs(Rx^2-2\,Rx\,Tx+Tx^2+Ry^2-2\,Ry\,Ty+Ty^2+Rz^2-2\,Rz\,Tz+Tz^2=R2, \%)$:
$factor(\%)$;

$$\frac{2\,(Ry-Ty)\,D1}{(\sqrt{R2}+D1)^2\,(-\sqrt{R2}+D1)^2} \tag{3.17}$$

$diff(SSSPZ(Rx, Ry, Rz, Tx, Ty, Tz, D1), Rz)$:
$subs(Rx^2-2\,Rx\,Tx+Tx^2+Ry^2-2\,Ry\,Ty+Ty^2+Rz^2-2\,Rz\,Tz+Tz^2=R2, \%)$:
$factor(\%)$;

$$\frac{2\,(Rz-Tz)\,D1}{(\sqrt{R2}+D1)^2\,(-\sqrt{R2}+D1)^2} \tag{3.18}$$

Derived projection operators

The projection operators are

X = R - T

normalization of X is sqrt(1-X[3]*X[3])

Y = [-X[2], X[1], 0]

Z = X \times Y

In the manuscript, they are R = X, u = Y and w = Z

Derivatives of projection operator X

x[1]
$diff\left(\frac{Rx-Tx}{\mathrm{sqrt}((Rx-Tx)^2+(Ry-Ty)^2+(Rz-Tz)^2)}, Rx\right)$:
$subs(Rx^2-2\,Rx\,Tx+Tx^2+Ry^2-2\,Ry\,Ty+Ty^2+Rz^2-2\,Rz\,Tz+Tz^2=R2, \%)$:

$factor(\%);$

$$\frac{R2 - Rx^2 + 2\,Rx\,Tx - Tx^2}{R2^{3/2}} \quad \textbf{(4.1.1)}$$

$diff\left(\frac{Rx - Tx}{\mathrm{sqrt}\left((Rx - Tx)^2 + (Ry - Ty)^2 + (Rz - Tz)^2\right)}, Ry\right):$
$subs\left(Rx^2 - 2\,Rx\,Tx + Tx^2 + Ry^2 - 2\,Ry\,Ty + Ty^2 + Rz^2 - 2\,Rz\,Tz + Tz^2 = R2, \%\right):$
$factor(\%);$

$$-\frac{(Rx - Tx)\,(Ry - Ty)}{R2^{3/2}} \quad \textbf{(4.1.2)}$$

$diff\left(\frac{Rx - Tx}{\mathrm{sqrt}\left((Rx - Tx)^2 + (Ry - Ty)^2 + (Rz - Tz)^2\right)}, Rz\right):$
$subs\left(Rx^2 - 2\,Rx\,Tx + Tx^2 + Ry^2 - 2\,Ry\,Ty + Ty^2 + Rz^2 - 2\,Rz\,Tz + Tz^2 = R2, \%\right):$
$factor(\%);$

$$-\frac{(Rx - Tx)\,(Rz - Tz)}{R2^{3/2}} \quad \textbf{(4.1.3)}$$

x[2]

$diff\left(\frac{Ry - Ty}{\mathrm{sqrt}\left((Rx - Tx)^2 + (Ry - Ty)^2 + (Rz - Tz)^2\right)}, Rx\right):$
$subs\left(Rx^2 - 2\,Rx\,Tx + Tx^2 + Ry^2 - 2\,Ry\,Ty + Ty^2 + Rz^2 - 2\,Rz\,Tz + Tz^2 = R2, \%\right):$
$factor(\%);$

$$-\frac{(Rx - Tx)\,(Ry - Ty)}{R2^{3/2}} \quad \textbf{(4.1.4)}$$

$diff\left(\frac{Ry - Ty}{\mathrm{sqrt}\left((Rx - Tx)^2 + (Ry - Ty)^2 + (Rz - Tz)^2\right)}, Ry\right):$
$subs\left(Rx^2 - 2\,Rx\,Tx + Tx^2 + Ry^2 - 2\,Ry\,Ty + Ty^2 + Rz^2 - 2\,Rz\,Tz + Tz^2 = R2, \%\right):$
$factor(\%);$

$$\frac{R2 - Ry^2 + 2\,Ry\,Ty - Ty^2}{R2^{3/2}} \quad \textbf{(4.1.5)}$$

$diff\left(\frac{Ry - Ty}{\mathrm{sqrt}\left((Rx - Tx)^2 + (Ry - Ty)^2 + (Rz - Tz)^2\right)}, Rz\right):$
$subs\left(Rx^2 - 2\,Rx\,Tx + Tx^2 + Ry^2 - 2\,Ry\,Ty + Ty^2 + Rz^2 - 2\,Rz\,Tz + Tz^2 = R2, \%\right):$
$factor(\%);$

$$-\frac{(Ry - Ty)\,(Rz - Tz)}{R2^{3/2}} \quad \textbf{(4.1.6)}$$

x[3]

$diff\left(\frac{Rz - Tz}{\mathrm{sqrt}\left((Rx - Tx)^2 + (Ry - Ty)^2 + (Rz - Tz)^2\right)}, Rx\right):$
$subs\left(Rx^2 - 2\,Rx\,Tx + Tx^2 + Ry^2 - 2\,Ry\,Ty + Ty^2 + Rz^2 - 2\,Rz\,Tz + Tz^2 = R2, \%\right):$
$factor(\%);$

$$-\frac{(Rx - Tx)\,(Rz - Tz)}{R2^{3/2}} \quad \textbf{(4.1.7)}$$

$$diff\left(\frac{Rz - Tz}{\mathrm{sqrt}\left((Rx - Tx)^2 + (Ry - Ty)^2 + (Rz - Tz)^2\right)}, Ry\right):$$
$$subs\left(Rx^2 - 2\,Rx\,Tx + Tx^2 + Ry^2 - 2\,Ry\,Ty + Ty^2 + Rz^2 - 2\,Rz\,Tz + Tz^2 = R2, \%\right):$$
$$factor(\%);$$

$$-\frac{(Ry - Ty)\,(Rz - Tz)}{R2^{3/2}} \quad (4.1.8)$$

$$diff\left(\frac{Rz - Tz}{\mathrm{sqrt}\left((Rx - Tx)^2 + (Ry - Ty)^2 + (Rz - Tz)^2\right)}, Rz\right):$$
$$subs\left(Rx^2 - 2\,Rx\,Tx + Tx^2 + Ry^2 - 2\,Ry\,Ty + Ty^2 + Rz^2 - 2\,Rz\,Tz + Tz^2 = R2, \%\right):$$
$$factor(\%);$$

$$\frac{R2 - Rz^2 + 2\,Rz\,Tz - Tz^2}{R2^{3/2}} \quad (4.1.9)$$

x[1]*x[1]

$$diff\left(\left(\frac{Rx - Tx}{\mathrm{sqrt}\left((Rx - Tx)^2 + (Ry - Ty)^2 + (Rz - Tz)^2\right)}\right)^2, Rx\right):$$
$$subs\left(Rx^2 - 2\,Rx\,Tx + Tx^2 + Ry^2 - 2\,Ry\,Ty + Ty^2 + Rz^2 - 2\,Rz\,Tz + Tz^2 = R2, \%\right):$$
$$factor(\%);$$

$$\frac{2\,(Rx - Tx)\,\left(R2 - Rx^2 + 2\,Rx\,Tx - Tx^2\right)}{R2^2} \quad (4.1.10)$$

$$diff\left(\left(\frac{Rx - Tx}{\mathrm{sqrt}\left((Rx - Tx)^2 + (Ry - Ty)^2 + (Rz - Tz)^2\right)}\right)^2, Ry\right):$$
$$subs\left(Rx^2 - 2\,Rx\,Tx + Tx^2 + Ry^2 - 2\,Ry\,Ty + Ty^2 + Rz^2 - 2\,Rz\,Tz + Tz^2 = R2, \%\right):$$
$$factor(\%);$$

$$-\frac{2\,(Rx - Tx)^2\,(Ry - Ty)}{R2^2} \quad (4.1.11)$$

$$diff\left(\left(\frac{Rx - Tx}{\mathrm{sqrt}\left((Rx - Tx)^2 + (Ry - Ty)^2 + (Rz - Tz)^2\right)}\right)^2, Rz\right):$$
$$subs\left(Rx^2 - 2\,Rx\,Tx + Tx^2 + Ry^2 - 2\,Ry\,Ty + Ty^2 + Rz^2 - 2\,Rz\,Tz + Tz^2 = R2, \%\right):$$
$$factor(\%);$$

$$-\frac{2\,(Rx - Tx)^2\,(Rz - Tz)}{R2^2} \quad (4.1.12)$$

x[2]*x[2]

$$diff\left(\left(\frac{Ry - Ty}{\mathrm{sqrt}\left((Rx - Tx)^2 + (Ry - Ty)^2 + (Rz - Tz)^2\right)}\right)^2, Rx\right):$$
$$subs\left(Rx^2 - 2\,Rx\,Tx + Tx^2 + Ry^2 - 2\,Ry\,Ty + Ty^2 + Rz^2 - 2\,Rz\,Tz + Tz^2 = R2, \%\right):$$
$$factor(\%);$$

$$-\frac{2\,(Ry - Ty)^2\,(Rx - Tx)}{R2^2} \quad (4.1.13)$$

$$diff\left(\left(\frac{Ry - Ty}{\mathrm{sqrt}\left((Rx - Tx)^2 + (Ry - Ty)^2 + (Rz - Tz)^2\right)}\right)^2, Ry\right):$$
$$subs\left(Rx^2 - 2\,Rx\,Tx + Tx^2 + Ry^2 - 2\,Ry\,Ty + Ty^2 + Rz^2 - 2\,Rz\,Tz + Tz^2 = R2, \%\right):$$
$$factor(\%);$$

$$\frac{2\,(Ry - Ty)\,\left(R2 - Ry^2 + 2\,Ry\,Ty - Ty^2\right)}{R2^2} \quad (4.1.14)$$

$$diff\left(\left(\frac{Ry - Ty}{\mathrm{sqrt}\left((Rx - Tx)^2 + (Ry - Ty)^2 + (Rz - Tz)^2\right)}\right)^2, Rz\right):$$
$$subs\left(Rx^2 - 2\,Rx\,Tx + Tx^2 + Ry^2 - 2\,Ry\,Ty + Ty^2 + Rz^2 - 2\,Rz\,Tz + Tz^2 = R2, \%\right):$$
$$factor(\%);$$

$$-\frac{2\,(Ry - Ty)^2\,(Rz - Tz)}{R2^2} \quad (4.1.15)$$

x[3]*x[3]

$$diff\left(\left(\frac{Rz - Tz}{\mathrm{sqrt}\left((Rx - Tx)^2 + (Ry - Ty)^2 + (Rz - Tz)^2\right)}\right)^2, Rx\right):$$
$$subs\left(Rx^2 - 2\,Rx\,Tx + Tx^2 + Ry^2 - 2\,Ry\,Ty + Ty^2 + Rz^2 - 2\,Rz\,Tz + Tz^2 = R2, \%\right):$$
$$factor(\%);$$

$$-\frac{2\,(Rz - Tz)^2\,(Rx - Tx)}{R2^2} \quad (4.1.16)$$

$$diff\left(\left(\frac{Rz - Tz}{\mathrm{sqrt}\left((Rx - Tx)^2 + (Ry - Ty)^2 + (Rz - Tz)^2\right)}\right)^2, Ry\right):$$
$$subs\left(Rx^2 - 2\,Rx\,Tx + Tx^2 + Ry^2 - 2\,Ry\,Ty + Ty^2 + Rz^2 - 2\,Rz\,Tz + Tz^2 = R2, \%\right):$$
$$factor(\%);$$

$$-\frac{2\,(Rz - Tz)^2\,(Ry - Ty)}{R2^2} \quad (4.1.17)$$

$$diff\left(\left(\frac{Rz - Tz}{\mathrm{sqrt}\left((Rx - Tx)^2 + (Ry - Ty)^2 + (Rz - Tz)^2\right)}\right)^2, Rz\right):$$
$$subs\left(Rx^2 - 2\,Rx\,Tx + Tx^2 + Ry^2 - 2\,Ry\,Ty + Ty^2 + Rz^2 - 2\,Rz\,Tz + Tz^2 = R2, \%\right):$$
$$factor(\%);$$

$$\frac{2\,(Rz - Tz)\,\left(R2 - Rz^2 + 2\,Rz\,Tz - Tz^2\right)}{R2^2} \quad (4.1.18)$$

x[2]*x[1]

$$diff\left(\frac{Ry - Ty}{\mathrm{sqrt}\left((Rx - Tx)^2 + (Ry - Ty)^2 + (Rz - Tz)^2\right)} \cdot \frac{Rx - Tx}{\mathrm{sqrt}\left((Rx - Tx)^2 + (Ry - Ty)^2 + (Rz - Tz)^2\right)}, Rx\right);$$

$$-\frac{(Ry - Ty)\,(Rx - Tx)\,(2\,Rx - 2\,Tx)}{\left(Rx^2 - 2\,Rx\,Tx + Tx^2 + Ry^2 - 2\,Ry\,Ty + Ty^2 + Rz^2 - 2\,Rz\,Tz + Tz^2\right)^2} + \frac{Ry - Ty}{Rx^2 - 2\,Rx\,Tx + Tx^2 + Ry^2 - 2\,Ry\,Ty + Ty^2 + Rz^2 - 2\,Rz\,Tz + Tz^2} \quad (4.1.19)$$

$$diff\left(\frac{Ry - Ty}{\operatorname{sqrt}\left((Rx-Tx)^2+(Ry-Ty)^2+(Rz-Tz)^2\right)} \cdot \frac{Rx - Tx}{\operatorname{sqrt}\left((Rx-Tx)^2+(Ry-Ty)^2+(Rz-Tz)^2\right)}, Ry\right);$$

$$\frac{Rx-Tx}{Rx^2-2\,Rx\,Tx+Tx^2+Ry^2-2\,Ry\,Ty+Ty^2+Rz^2-2\,Rz\,Tz+Tz^2} - \frac{(Ry-Ty)\,(Rx-Tx)\,(2\,Ry-2\,Ty)}{\left(Rx^2-2\,Rx\,Tx+Tx^2+Ry^2-2\,Ry\,Ty+Ty^2+Rz^2-2\,Rz\,Tz+Tz^2\right)^2} \tag{4.1.20}$$

$$diff\left(\frac{Ry - Ty}{\operatorname{sqrt}\left((Rx-Tx)^2+(Ry-Ty)^2+(Rz-Tz)^2\right)} \cdot \frac{Rx - Tx}{\operatorname{sqrt}\left((Rx-Tx)^2+(Ry-Ty)^2+(Rz-Tz)^2\right)}, Rz\right);$$

$$-\frac{(Ry-Ty)\,(Rx-Tx)\,(2\,Rz-2\,Tz)}{\left(Rx^2-2\,Rx\,Tx+Tx^2+Ry^2-2\,Ry\,Ty+Ty^2+Rz^2-2\,Rz\,Tz+Tz^2\right)^2} \tag{4.1.21}$$

x[3]*x[1]

$$diff\left(\frac{Rz - Tz}{\operatorname{sqrt}\left((Rx-Tx)^2+(Ry-Ty)^2+(Rz-Tz)^2\right)} \cdot \frac{Rx - Tx}{\operatorname{sqrt}\left((Rx-Tx)^2+(Ry-Ty)^2+(Rz-Tz)^2\right)}, Rx\right);$$

$$-\frac{(Rz-Tz)\,(Rx-Tx)\,(2\,Rx-2\,Tx)}{\left(Rx^2-2\,Rx\,Tx+Tx^2+Ry^2-2\,Ry\,Ty+Ty^2+Rz^2-2\,Rz\,Tz+Tz^2\right)^2} + \frac{Rz-Tz}{Rx^2-2\,Rx\,Tx+Tx^2+Ry^2-2\,Ry\,Ty+Ty^2+Rz^2-2\,Rz\,Tz+Tz^2} \tag{4.1.22}$$

$$diff\left(\frac{Rz - Tz}{\operatorname{sqrt}\left((Rx-Tx)^2+(Ry-Ty)^2+(Rz-Tz)^2\right)} \cdot \frac{Rx - Tx}{\operatorname{sqrt}\left((Rx-Tx)^2+(Ry-Ty)^2+(Rz-Tz)^2\right)}, Ry\right);$$

$$-\frac{(Rz-Tz)\,(Rx-Tx)\,(2\,Ry-2\,Ty)}{\left(Rx^2-2\,Rx\,Tx+Tx^2+Ry^2-2\,Ry\,Ty+Ty^2+Rz^2-2\,Rz\,Tz+Tz^2\right)^2} \tag{4.1.23}$$

$$diff\left(\frac{Rz - Tz}{\operatorname{sqrt}\left((Rx-Tx)^2+(Ry-Ty)^2+(Rz-Tz)^2\right)} \cdot \frac{Rx - Tx}{\operatorname{sqrt}\left((Rx-Tx)^2+(Ry-Ty)^2+(Rz-Tz)^2\right)}, Rz\right);$$

$$\frac{Rx-Tx}{Rx^2-2\,Rx\,Tx+Tx^2+Ry^2-2\,Ry\,Ty+Ty^2+Rz^2-2\,Rz\,Tz+Tz^2} - \frac{(Rz-Tz)\,(Rx-Tx)\,(2\,Rz-2\,Tz)}{\left(Rx^2-2\,Rx\,Tx+Tx^2+Ry^2-2\,Ry\,Ty+Ty^2+Rz^2-2\,Rz\,Tz+Tz^2\right)^2} \tag{4.1.24}$$

x[3]*x[2]

$$diff\left(\frac{Rz - Tz}{\operatorname{sqrt}\left((Rx-Tx)^2+(Ry-Ty)^2+(Rz-Tz)^2\right)}\right.$$

$$\cdot \frac{Ry - Ty}{\mathrm{sqrt}\left((Rx - Tx)^2 + (Ry - Ty)^2 + (Rz - Tz)^2\right)}, Rx\Bigg);$$

$$-\frac{(Rz - Tz)\,(Ry - Ty)\,(2\,Rx - 2\,Tx)}{\left(Rx^2 - 2\,Rx\,Tx + Tx^2 + Ry^2 - 2\,Ry\,Ty + Ty^2 + Rz^2 - 2\,Rz\,Tz + Tz^2\right)^2} \tag{4.1.25}$$

$$diff\left(\frac{Rz - Tz}{\mathrm{sqrt}\left((Rx - Tx)^2 + (Ry - Ty)^2 + (Rz - Tz)^2\right)} \cdot \frac{Ry - Ty}{\mathrm{sqrt}\left((Rx - Tx)^2 + (Ry - Ty)^2 + (Rz - Tz)^2\right)}, Ry\right);$$

$$-\frac{(Rz - Tz)\,(Ry - Ty)\,(2\,Ry - 2\,Ty)}{\left(Rx^2 - 2\,Rx\,Tx + Tx^2 + Ry^2 - 2\,Ry\,Ty + Ty^2 + Rz^2 - 2\,Rz\,Tz + Tz^2\right)^2} + \frac{Rz - Tz}{Rx^2 - 2\,Rx\,Tx + Tx^2 + Ry^2 - 2\,Ry\,Ty + Ty^2 + Rz^2 - 2\,Rz\,Tz + Tz^2} \tag{4.1.26}$$

$$diff\left(\frac{Rz - Tz}{\mathrm{sqrt}\left((Rx - Tx)^2 + (Ry - Ty)^2 + (Rz - Tz)^2\right)} \cdot \frac{Ry - Ty}{\mathrm{sqrt}\left((Rx - Tx)^2 + (Ry - Ty)^2 + (Rz - Tz)^2\right)}, Rz\right);$$

$$\frac{Ry - Ty}{Rx^2 - 2\,Rx\,Tx + Tx^2 + Ry^2 - 2\,Ry\,Ty + Ty^2 + Rz^2 - 2\,Rz\,Tz + Tz^2} - \frac{(Rz - Tz)\,(Ry - Ty)\,(2\,Rz - 2\,Tz)}{\left(Rx^2 - 2\,Rx\,Tx + Tx^2 + Ry^2 - 2\,Ry\,Ty + Ty^2 + Rz^2 - 2\,Rz\,Tz + Tz^2\right)^2} \tag{4.1.27}$$

Derivatives of projection operator Y

y[1]*y[1] = x[2]*x[2] / (1-x[3]*x[3])

$$Y11_x := (Rx, Ry, Rz, Tx, Ty, Tz) \rightarrow diff\left(Y_1(Rx, Ry, Rz, Tx, Ty, Tz) \cdot Y_1(Rx, Ry, Rz, Tx, Ty, Tz), Rx\right):$$

$$Y11_x(Rx, Ry, Rz, Tx, Ty, Tz);$$

$$-\frac{(Ry - Ty)^2\,(2\,Rx - 2\,Tx)}{\left(Rx^2 - 2\,Rx\,Tx + Tx^2 + Ry^2 - 2\,Ry\,Ty + Ty^2\right)^2} \tag{4.2.1}$$

$$subs\left(Rx^2 - 2\,Rx\,Tx + Tx^2 + Ry^2 - 2\,Ry\,Ty + Ty^2 = RY2, \%\right);$$

$$-\frac{(Ry - Ty)^2\,(2\,Rx - 2\,Tx)}{RY2^2} \tag{4.2.2}$$

$$Y11_y := (Rx, Ry, Rz, Tx, Ty, Tz) \rightarrow diff\left(Y_1(Rx, Ry, Rz, Tx, Ty, Tz) \cdot Y_1(Rx, Ry, Rz, Tx, Ty, Tz), Ry\right):$$

$$Y11_y(Rx, Ry, Rz, Tx, Ty, Tz);$$

$$\frac{2\,(Ry - Ty)}{Rx^2 - 2\,Rx\,Tx + Tx^2 + Ry^2 - 2\,Ry\,Ty + Ty^2} - \frac{(Ry - Ty)^2\,(2\,Ry - 2\,Ty)}{\left(Rx^2 - 2\,Rx\,Tx + Tx^2 + Ry^2 - 2\,Ry\,Ty + Ty^2\right)^2} \tag{4.2.3}$$

(4.2.4)

$subs\left(Rx^2 - 2\,Rx\,Tx + Tx^2 + Ry^2 - 2\,Ry\,Ty + Ty^2 = RY2, \%\right);$

$$\frac{2\,(Ry - Ty)}{RY2} - \frac{(Ry - Ty)^2\,(2\,Ry - 2\,Ty)}{RY2^2} \quad (4.2.5)$$

$Y11_z := (Rx, Ry, Rz, Tx, Ty, Tz) \rightarrow diff\left(Y_1(Rx, Ry, Rz, Tx, Ty, Tz) \cdot Y_1(Rx, Ry, Rz, Tx, Ty, Tz), Rz\right):$

$Y11_z(Rx, Ry, Rz, Tx, Ty, Tz);$

$$0 \quad (4.2.6)$$

(4.2.7)

$subs\left(Rx^2 - 2\,Rx\,Tx + Tx^2 + Ry^2 - 2\,Ry\,Ty + Ty^2 = RY2, \%\right);$

$$0 \quad (4.2.8)$$

y[2]*y[1] = -x[1]*x[2] / (1-x[3]*x[3])

$Y21_x := (Rx, Ry, Rz, Tx, Ty, Tz) \rightarrow diff\left(Y_2(Rx, Ry, Rz, Tx, Ty, Tz) \cdot Y_1(Rx, Ry, Rz, Tx, Ty, Tz), Rx\right):$

$Y21_x(Rx, Ry, Rz, Tx, Ty, Tz);$

$$-\frac{Ry - Ty}{Rx^2 - 2\,Rx\,Tx + Tx^2 + Ry^2 - 2\,Ry\,Ty + Ty^2} + \frac{(Rx - Tx)\,(Ry - Ty)\,(2\,Rx - 2\,Tx)}{\left(Rx^2 - 2\,Rx\,Tx + Tx^2 + Ry^2 - 2\,Ry\,Ty + Ty^2\right)^2} \quad (4.2.9)$$

(4.2.10)

$subs\left(Rx^2 - 2\,Rx\,Tx + Tx^2 + Ry^2 - 2\,Ry\,Ty + Ty^2 = RY2, \%\right);$

$$-\frac{Ry - Ty}{RY2} + \frac{(Rx - Tx)\,(Ry - Ty)\,(2\,Rx - 2\,Tx)}{RY2^2} \quad (4.2.11)$$

$Y21_y := (Rx, Ry, Rz, Tx, Ty, Tz) \rightarrow diff\left(Y_2(Rx, Ry, Rz, Tx, Ty, Tz) \cdot Y_1(Rx, Ry, Rz, Tx, Ty, Tz), Ry\right):$

$Y21_y(Rx, Ry, Rz, Tx, Ty, Tz);$

$$\frac{(Rx - Tx)\,(Ry - Ty)\,(2\,Ry - 2\,Ty)}{\left(Rx^2 - 2\,Rx\,Tx + Tx^2 + Ry^2 - 2\,Ry\,Ty + Ty^2\right)^2} - \frac{Rx - Tx}{Rx^2 - 2\,Rx\,Tx + Tx^2 + Ry^2 - 2\,Ry\,Ty + Ty^2} \quad (4.2.12)$$

(4.2.13)

$subs\left(Rx^2 - 2\,Rx\,Tx + Tx^2 + Ry^2 - 2\,Ry\,Ty + Ty^2 = RY2, \%\right);$

$$\frac{(Rx - Tx)\,(Ry - Ty)\,(2\,Ry - 2\,Ty)}{RY2^2} - \frac{Rx - Tx}{RY2} \quad (4.2.14)$$

$Y21_z := (Rx, Ry, Rz, Tx, Ty, Tz) \rightarrow diff\left(Y_2(Rx, Ry, Rz, Tx, Ty, Tz) \cdot Y_1(Rx, Ry, Rz, Tx, Ty, Tz), Rz\right):$

$Y21_z(Rx, Ry, Rz, Tx, Ty, Tz);$

$$0 \quad (4.2.15)$$

(4.2.16)

$subs\left(Rx^2 - 2\,Rx\,Tx + Tx^2 + Ry^2 - 2\,Ry\,Ty + Ty^2 = RY2, \%\right);$

$$0 \quad (4.2.17)$$

y[2]*y[2] = x[1]*x[1] / (1-x[3]*x[3])

$Y22_x := (Rx, Ry, Rz, Tx, Ty, Tz) \rightarrow diff\left(Y_2(Rx, Ry, Rz, Tx, Ty, Tz) \cdot Y_2(Rx, Ry, Rz, Tx, Ty, Tz), Rx\right):$

$Y22_x(Rx, Ry, Rz, Tx, Ty, Tz);$

$$\frac{2\,(Rx - Tx)}{Rx^2 - 2\,Rx\,Tx + Tx^2 + Ry^2 - 2\,Ry\,Ty + Ty^2} - \frac{(Rx - Tx)^2\,(2\,Rx - 2\,Tx)}{\left(Rx^2 - 2\,Rx\,Tx + Tx^2 + Ry^2 - 2\,Ry\,Ty + Ty^2\right)^2} \quad (4.2.18)$$

$\#eval(\%, \{Rx = -16.692327595, Ry = 2.053546334, Rz = 4.935321773, Tx = -16.319510615, Ty = 3.200959013, Tz = 8.115683388, D2 = 0.512573800\});$

$subs\left(Rx^2 - 2\,Rx\,Tx + Tx^2 + Ry^2 - 2\,Ry\,Ty + Ty^2 = RY2, \%\right);$

$$\frac{2\,(Rx - Tx)}{RY2} - \frac{(Rx - Tx)^2\,(2\,Rx - 2\,Tx)}{RY2^2} \quad (4.2.19)$$

$Y22_y := (Rx, Ry, Rz, Tx, Ty, Tz) \rightarrow diff\left(Y_2(Rx, Ry, Rz, Tx, Ty, Tz) \cdot Y_2(Rx, Ry, Rz, Tx, Ty, Tz), Ry\right):$

$Y22_y(Rx, Ry, Rz, Tx, Ty, Tz);$

$$-\frac{(Rx - Tx)^2\,(2\,Ry - 2\,Ty)}{\left(Rx^2 - 2\,Rx\,Tx + Tx^2 + Ry^2 - 2\,Ry\,Ty + Ty^2\right)^2} \quad (4.2.20)$$

$\#eval(\%, \{Rx = -16.692327595, Ry = 2.053546334, Rz = 4.935321773, Tx = -16.319510615, Ty = 3.200959013, Tz = 8.115683388, D2 = 0.512573800\});$

$subs\left(Rx^2 - 2\,Rx\,Tx + Tx^2 + Ry^2 - 2\,Ry\,Ty + Ty^2 = RY2, \%\right);$

$$-\frac{(Rx - Tx)^2\,(2\,Ry - 2\,Ty)}{RY2^2} \quad (4.2.21)$$

$Y22_z := (Rx, Ry, Rz, Tx, Ty, Tz) \rightarrow diff\left(Y_2(Rx, Ry, Rz, Tx, Ty, Tz) \cdot Y_2(Rx, Ry, Rz, Tx, Ty, Tz), Rz\right):$

(4.2.22)

$subs\left(Rx^2 - 2\,Rx\,Tx + Tx^2 + Ry^2 - 2\,Ry\,Ty + Ty^2 = RY2, \%\right);$

$$(Rx, Ry, Rz, Tx, Ty, Tz) \rightarrow \frac{\partial}{\partial Rz}\left(Y_2(Rx, Ry, Rz, Tx, Ty, Tz)^2\right) \quad (4.2.23)$$

Derivatives of projection operator Z

z[1]*z[1]

$Z11_x := (Rx, Ry, Rz, Tx, Ty, Tz) \rightarrow diff\left(Z_1(Rx, Ry, Rz, Tx, Ty, Tz) \cdot Z_1(Rx, Ry, Rz, Tx, Ty, Tz), Rx\right):$

$Z11_x(Rx, Ry, Rz, Tx, Ty, Tz);$

$\left(2\,(Rx - Tx)\,(Rz - Tz)^2\right) / \left(\left(Rx^2 - 2\,Rx\,Tx + Tx^2 + Ry^2 - 2\,Ry\,Ty + Ty^2 + Rz^2\right.\right.$

$$-2\,Rz\,Tz + Tz^2)\,(Rx^2 - 2\,Rx\,Tx + Tx^2 + Ry^2 - 2\,Ry\,Ty + Ty^2)\,) - (\,(Rx - Tx)^2\,(Rz - Tz)^2\,(2\,Rx -$$
$$+ Ty^2)\,) - (\,(Rx - Tx)^2\,(Rz - Tz)^2\,(2\,Rx - 2\,Tx)\,) \Big/ (\,(Rx^2 - 2\,Rx\,Tx + Tx^2$$
$$+ Ry^2 - 2\,Ry\,Ty + Ty^2 + Rz^2 - 2\,Rz\,Tz + Tz^2)\,(Rx^2 - 2\,Rx\,Tx + Tx^2 + Ry^2$$
$$- 2\,Ry\,Ty + Ty^2)^2)$$

$\#eval(\%, \{Rx = -16.692327595, Ry = 2.053546334, Rz = 4.935321773, Tx = -16.319510615, Ty = 3.200959013, Tz = 8.115683388, D2 = 0.512573800\});$

$subs(Rx^2 - 2\,Rx\,Tx + Tx^2 + Ry^2 - 2\,Ry\,Ty + Ty^2 + Rz^2 - 2\,Rz\,Tz + Tz^2 = R2, \%)$:

$subs(Rx^2 - 2\,Rx\,Tx + Tx^2 + Ry^2 - 2\,Ry\,Ty + Ty^2 = RY2, \%);$

$$\frac{2\,(Rx - Tx)\,(Rz - Tz)^2}{R2\,RY2} - \frac{(Rx - Tx)^2\,(Rz - Tz)^2\,(2\,Rx - 2\,Tx)}{R2^2\,RY2} - \frac{(Rx - Tx)^2\,(Rz - Tz)^2\,(2\,Rx - 2\,Tx)}{R2\,RY2^2} \quad \textbf{(4.3.2)}$$

$Z11_y := (Rx, Ry, Rz, Tx, Ty, Tz) \rightarrow diff(Z_1(Rx, Ry, Rz, Tx, Ty, Tz) \cdot Z_1(Rx, Ry, Rz, Tx, Ty, Tz), Ry)$:

$Z11_y(Rx, Ry, Rz, Tx, Ty, Tz);$

$$-(\,(Rx - Tx)^2\,(Rz - Tz)^2\,(2\,Ry - 2\,Ty)\,) \Big/ (\,(Rx^2 - 2\,Rx\,Tx + Tx^2 + Ry^2 - 2\,Ry\,Ty + Ty^2$$
$$+ Rz^2 - 2\,Rz\,Tz + Tz^2)^2\,(Rx^2 - 2\,Rx\,Tx + Tx^2 + Ry^2 - 2\,Ry\,Ty + Ty^2)\,)$$
$$- (\,(Rx - Tx)^2\,(Rz - Tz)^2\,(2\,Ry - 2\,Ty)\,) \Big/ (\,(Rx^2 - 2\,Rx\,Tx + Tx^2 + Ry^2 - 2\,Ry\,Ty + Ty^2 + Rz^2 - 2$$
$$+ Ty^2)^2)$$

(4.3.4)

$subs(Rx^2 - 2\,Rx\,Tx + Tx^2 + Ry^2 - 2\,Ry\,Ty + Ty^2 + Rz^2 - 2\,Rz\,Tz + Tz^2 = R2, \%)$:

$subs(Rx^2 - 2\,Rx\,Tx + Tx^2 + Ry^2 - 2\,Ry\,Ty + Ty^2 = RY2, \%);$

$$-\frac{(Rx - Tx)^2\,(Rz - Tz)^2\,(2\,Ry - 2\,Ty)}{R2^2\,RY2} - \frac{(Rx - Tx)^2\,(Rz - Tz)^2\,(2\,Ry - 2\,Ty)}{R2\,RY2^2} \quad \textbf{(4.3.5)}$$

$Z11_z := (Rx, Ry, Rz, Tx, Ty, Tz) \rightarrow diff(Z_1(Rx, Ry, Rz, Tx, Ty, Tz) \cdot Z_1(Rx, Ry, Rz, Tx, Ty, Tz), Rz)$:

$Z11_z(Rx, Ry, Rz, Tx, Ty, Tz);$

$$(2\,(Rx - Tx)^2\,(Rz - Tz)\,) \Big/ (\,(Rx^2 - 2\,Rx\,Tx + Tx^2 + Ry^2 - 2\,Ry\,Ty + Ty^2 + Rz^2$$
$$- 2\,Rz\,Tz + Tz^2)\,(Rx^2 - 2\,Rx\,Tx + Tx^2 + Ry^2 - 2\,Ry\,Ty + Ty^2)\,) - (\,(Rx - Tx)^2\,(Rz - Tz)^2\,(2\,Rz -$$
$$+ Ty^2)\,)$$

$subs(Rx^2 - 2\,Rx\,Tx + Tx^2 + Ry^2 - 2\,Ry\,Ty + Ty^2 + Rz^2 - 2\,Rz\,Tz + Tz^2 = R2, \%)$:

$subs(Rx^2 - 2\,Rx\,Tx + Tx^2 + Ry^2 - 2\,Ry\,Ty + Ty^2 = RY2, \%);$

$$\frac{2\,(Rx - Tx)^2\,(Rz - Tz)}{R2\,RY2} - \frac{(Rx - Tx)^2\,(Rz - Tz)^2\,(2\,Rz - 2\,Tz)}{R2^2\,RY2} \quad \textbf{(4.3.7)}$$

z[2]*z[2]

$Z22_x := (Rx, Ry, Rz, Tx, Ty, Tz) \rightarrow diff(Z_2(Rx, Ry, Rz, Tx, Ty, Tz) \cdot Z_2(Rx, Ry, Rz, Tx, Ty, Tz),$

$Rx\big)$:

$Z22_x(Rx, Ry, Rz, Tx, Ty, Tz);$

$$-\big((Ry-Ty)^2\,(Rz-Tz)^2\,(2\,Rx-2\,Tx)\big)\Big/\Big(\big(Rx^2-2\,Rx\,Tx+Tx^2+Ry^2-2\,Ry\,Ty+Ty^2+Rz^2-2\,Rz\,Tz+Tz^2\big)^2\,\big(Rx^2-2\,Rx\,Tx+Tx^2+Ry^2-2\,Ry\,Ty+Ty^2\big)\Big)-\big((Ry-Ty)^2\,(Rz-Tz)^2\,(2\,Rx-2\,Tx)\big)\Big/\Big(\big(Rx^2-2\,Rx\,Tx+Tx^2+Ry^2-2\,Ry\,Ty+Ty^2+Rz^2-2 \text{[illegible]} + Ty^2\big)^2\Big)$$

$subs\big(Rx^2-2\,Rx\,Tx+Tx^2+Ry^2-2\,Ry\,Ty+Ty^2+Rz^2-2\,Rz\,Tz+Tz^2=R2, \%\big)$:

$subs\big(Rx^2-2\,Rx\,Tx+Tx^2+Ry^2-2\,Ry\,Ty+Ty^2=RY2, \%\big);$

$$-\frac{(Ry-Ty)^2\,(Rz-Tz)^2\,(2\,Rx-2\,Tx)}{R2^2\,RY2}-\frac{(Ry-Ty)^2\,(Rz-Tz)^2\,(2\,Rx-2\,Tx)}{R2\,RY2^2} \qquad \textbf{(4.3.9)}$$

$Z22_y := (Rx, Ry, Rz, Tx, Ty, Tz)\rightarrow diff\big(Z_2(Rx, Ry, Rz, Tx, Ty, Tz)\cdot Z_2(Rx, Ry, Rz, Tx, Ty, Tz),$
$Ry\big)$:

$Z22_y(Rx, Ry, Rz, Tx, Ty, Tz);$

$$\big(2\,(Ry-Ty)\,(Rz-Tz)^2\big)\Big/\Big(\big(Rx^2-2\,Rx\,Tx+Tx^2+Ry^2-2\,Ry\,Ty+Ty^2+Rz^2-2\,Rz\,Tz+Tz^2\big)\,\big(Rx^2-2\,Rx\,Tx+Tx^2+Ry^2-2\,Ry\,Ty+Ty^2\big)\Big)-\big((Ry-Ty)^2\,(Rz-Tz)^2\,(2\,Ry-\text{[illegible]}+Ty^2)\big)-\big((Ry-Ty)^2\,(Rz-Tz)^2\,(2\,Ry-2\,Ty)\big)\Big/\Big(\big(Rx^2-2\,Rx\,Tx+Tx^2+Ry^2-2\,Ry\,Ty+Ty^2+Rz^2-2\,Rz\,Tz+Tz^2\big)\,\big(Rx^2-2\,Rx\,Tx+Tx^2+Ry^2-2\,Ry\,Ty+Ty^2\big)^2\Big)$$

(4.3.11)

$subs\big(Rx^2-2\,Rx\,Tx+Tx^2+Ry^2-2\,Ry\,Ty+Ty^2+Rz^2-2\,Rz\,Tz+Tz^2=R2, \%\big)$:

$subs\big(Rx^2-2\,Rx\,Tx+Tx^2+Ry^2-2\,Ry\,Ty+Ty^2=RY2, \%\big);$

$$\frac{2\,(Ry-Ty)\,(Rz-Tz)^2}{R2\,RY2}-\frac{(Ry-Ty)^2\,(Rz-Tz)^2\,(2\,Ry-2\,Ty)}{R2^2\,RY2}-\frac{(Ry-Ty)^2\,(Rz-Tz)^2\,(2\,Ry-2\,Ty)}{R2\,RY2^2} \qquad \textbf{(4.3.12)}$$

$Z22_z := (Rx, Ry, Rz, Tx, Ty, Tz)\rightarrow diff\big(Z_2(Rx, Ry, Rz, Tx, Ty, Tz)\cdot Z_2(Rx, Ry, Rz, Tx, Ty, Tz),$
$Rz\big)$:

$Z22_z(Rx, Ry, Rz, Tx, Ty, Tz);$

$$\big(2\,(Ry-Ty)^2\,(Rz-Tz)\big)\Big/\Big(\big(Rx^2-2\,Rx\,Tx+Tx^2+Ry^2-2\,Ry\,Ty+Ty^2+Rz^2-2\,Rz\,Tz+Tz^2\big)\,\big(Rx^2-2\,Rx\,Tx+Tx^2+Ry^2-2\,Ry\,Ty+Ty^2\big)\Big)-\big((Ry-Ty)^2\,(Rz-Tz)^2\,(2\,Rz-\text{[illegible]}+Ty^2)\big)$$

$subs\big(Rx^2-2\,Rx\,Tx+Tx^2+Ry^2-2\,Ry\,Ty+Ty^2+Rz^2-2\,Rz\,Tz+Tz^2=R2, \%\big)$:

$subs\big(Rx^2-2\,Rx\,Tx+Tx^2+Ry^2-2\,Ry\,Ty+Ty^2=RY2, \%\big);$

$$\frac{2\,(Ry-Ty)^2\,(Rz-Tz)}{R2\,RY2}-\frac{(Ry-Ty)^2\,(Rz-Tz)^2\,(2\,Rz-2\,Tz)}{R2^2\,RY2} \qquad \textbf{(4.3.14)}$$

z[3]*z[3] = 1-x[3]*x[3]

$Z33_x := (Rx, Ry, Rz, Tx, Ty, Tz) \rightarrow diff\left(Z_3(Rx, Ry, Rz, Tx, Ty, Tz) \cdot Z_3(Rx, Ry, Rz, Tx, Ty, Tz), Rx\right):$

$Z33_x(Rx, Ry, Rz, Tx, Ty, Tz);$

$$\left(2\left((Rx-Tx)^2+(Ry-Ty)^2\right)(2\,Rx-2\,Tx)\right)\Big/\left(\left(Rx^2-2\,Rx\,Tx+Tx^2+Ry^2-2\,Ry\,Ty+Ty^2+Rz^2-2\,Rz\,Tz+Tz^2\right)\left(Rx^2-2\,Rx\,Tx+Tx^2+Ry^2-2\,Ry\,Ty+Ty^2\right)\right)-\left(\left((Rx-Tx)^2+(Ry-Ty)^2\right)^2(2\,Rx-2\,Tx)\right)\Big/\left(\left(Rx^2-2\,Rx\,Tx+Tx^2+Ry^2-2\,Ry\,Ty+\right.\right.$$

$$\left.\left.-2\,Ry\,Ty+Ty^2\right)\right)-\left(\left((Rx-Tx)^2+(Ry-Ty)^2\right)^2(2\,Rx-2\,Tx)\right)\Big/\left(\left(Rx^2-2\,Rx\,Tx+Tx^2+Ry^2-2\,Ry\,Ty+Ty^2+Rz^2-2\,Rz\,Tz+Tz^2\right)\left(Rx^2-2\,Rx\,Tx+Tx^2+Ry^2-2\,Ry\,Ty+Ty^2\right)^2\right)$$

$subs\left(Rx^2-2\,Rx\,Tx+Tx^2+Ry^2-2\,Ry\,Ty+Ty^2+Rz^2-2\,Rz\,Tz+Tz^2=R2, \%\right):$

$subs\left(Rx^2-2\,Rx\,Tx+Tx^2+Ry^2-2\,Ry\,Ty+Ty^2=RY2, \%\right):$

$subs\left((Rx-Tx)^2+(Ry-Ty)^2=RY2, \%\right);$

$$\frac{2\,Rx-2\,Tx}{R2}-\frac{RY2\,(2\,Rx-2\,Tx)}{R2^2} \tag{4.3.16}$$

$Z33_y := (Rx, Ry, Rz, Tx, Ty, Tz) \rightarrow diff\left(Z_3(Rx, Ry, Rz, Tx, Ty, Tz) \cdot Z_3(Rx, Ry, Rz, Tx, Ty, Tz), Ry\right):$

$Z33_y(Rx, Ry, Rz, Tx, Ty, Tz);$

$$\left(2\left((Rx-Tx)^2+(Ry-Ty)^2\right)(2\,Ry-2\,Ty)\right)\Big/\left(\left(Rx^2-2\,Rx\,Tx+Tx^2+Ry^2-2\,Ry\,Ty+Ty^2+Rz^2-2\,Rz\,Tz+Tz^2\right)\left(Rx^2-2\,Rx\,Tx+Tx^2+Ry^2-2\,Ry\,Ty+Ty^2\right)\right)-\left(\left((Rx-Tx)^2+(Ry-Ty)^2\right)^2(2\,Ry-2\,Ty)\right)\Big/\left(\left(Rx^2-2\,Rx\,Tx+Tx^2+Ry^2-2\,Ry\,Ty+\right.\right.$$

$$\left.\left.-2\,Ry\,Ty+Ty^2\right)\right)-\left(\left((Rx-Tx)^2+(Ry-Ty)^2\right)^2(2\,Ry-2\,Ty)\right)\Big/\left(\left(Rx^2-2\,Rx\,Tx+Tx^2+Ry^2-2\,Ry\,Ty+Ty^2+Rz^2-2\,Rz\,Tz+Tz^2\right)\left(Rx^2-2\,Rx\,Tx+Tx^2+Ry^2-2\,Ry\,Ty+Ty^2\right)^2\right)$$

$\#eval(\%, \{Rx=-16.692327595, Ry=2.053546334, Rz=4.935321773, Tx=-16.319510615, Ty=3.200959013, Tz=8.115683388, D2=0.512573800\});$

$subs\left(Rx^2-2\,Rx\,Tx+Tx^2+Ry^2-2\,Ry\,Ty+Ty^2+Rz^2-2\,Rz\,Tz+Tz^2=R2, \%\right):$

$subs\left(Rx^2-2\,Rx\,Tx+Tx^2+Ry^2-2\,Ry\,Ty+Ty^2=RY2, \%\right):$

$subs\left((Rx-Tx)^2+(Ry-Ty)^2=RY2, \%\right);$

$$\frac{2\,Ry-2\,Ty}{R2}-\frac{RY2\,(2\,Ry-2\,Ty)}{R2^2} \tag{4.3.18}$$

$Z33_z := (Rx, Ry, Rz, Tx, Ty, Tz) \rightarrow diff\left(Z_3(Rx, Ry, Rz, Tx, Ty, Tz) \cdot Z_3(Rx, Ry, Rz, Tx, Ty, Tz), Rz\right):$

$Z33_z(Rx, Ry, Rz, Tx, Ty, Tz);$

$$-\left(\left((Rx-Tx)^2+(Ry-Ty)^2\right)^2(2\,Rz-2\,Tz)\right)\Big/\left(\left(Rx^2-2\,Rx\,Tx+Tx^2+Ry^2-2\,Ry\,Ty+Ty^2+Rz^2-2\,Rz\,Tz+Tz^2\right)^2\left(Rx^2-2\,Rx\,Tx+Tx^2+Ry^2-2\,Ry\,Ty+Ty^2\right)\right) \tag{4.3.19}$$

$subs(Rx^2 - 2\,Rx\,Tx + Tx^2 + Ry^2 - 2\,Ry\,Ty + Ty^2 + Rz^2 - 2\,Rz\,Tz + Tz^2 = R2, \%)$:
$subs(Rx^2 - 2\,Rx\,Tx + Tx^2 + Ry^2 - 2\,Ry\,Ty + Ty^2 = RY2, \%)$:
$subs((Rx - Tx)^2 + (Ry - Ty)^2 = RY2, \%)$;

$$-\frac{RY2\,(2\,Rz - 2\,Tz)}{R2^2} \qquad \textbf{(4.3.20)}$$

z[2]*z[1]

$Z21_x := (Rx, Ry, Rz, Tx, Ty, Tz) \rightarrow diff\left(Z_2(Rx, Ry, Rz, Tx, Ty, Tz) \cdot Z_1(Rx, Ry, Rz, Tx, Ty, Tz), Rx\right)$:
$Z21_x(Rx, Ry, Rz, Tx, Ty, Tz)$;

$$-\left((Ry - Ty)\,(Rz - Tz)^2\,(Rx - Tx)\,(2\,Rx - 2\,Tx)\right) \Big/ \left((Rx^2 - 2\,Rx\,Tx + Tx^2 + Ry^2 - 2\,Ry\,Ty + Ty^2 + Rz^2 - 2\,Rz\,Tz + Tz^2)^2\,(Rx^2 - 2\,Rx\,Tx + Tx^2 + Ry^2 - 2\,Ry\,Ty + Ty^2)\right) - \left((Ry - Ty)\,(Rz - Tz)^2\,(Rx - Tx)\,(2\,Rx - 2\,Tx)\right) \Big/ \left((Rx^2 - 2\,Rx\,Tx + Tx^2 + Ry^2 - 2\,Ry\,Ty + Ty^2 + Rz^2 - 2\,Rz\,Tz + Tz^2)\,(Rx^2 - 2\,Rx\,Tx + Tx^2 + Ry^2 - 2\,Ry\,Ty + Ty^2)^2\right) + \left((Ry - Ty)\,(Rz - Tz)^2\right) \Big/ \left((Rx^2 - 2\,Rx\,Tx + Tx^2 + Ry^2 - 2\,Ry\,Ty + Ty^2 + Rz^2 - 2\,Rz\,Tz + Tz^2)\,(Rx^2 - 2\,Rx\,Tx + Tx^2 + Ry^2 - 2\,Ry\,Ty + Ty^2)\right) \qquad \textbf{(4.3.21)}$$

(4.3.22)

$subs(Rx^2 - 2\,Rx\,Tx + Tx^2 + Ry^2 - 2\,Ry\,Ty + Ty^2 + Rz^2 - 2\,Rz\,Tz + Tz^2 = R2, \%)$:
$subs(Rx^2 - 2\,Rx\,Tx + Tx^2 + Ry^2 - 2\,Ry\,Ty + Ty^2 = RY2, \%)$:
$subs((Rx - Tx)^2 + (Ry - Ty)^2 = RY2, \%)$;

$$-\frac{(Ry - Ty)\,(Rz - Tz)^2\,(Rx - Tx)\,(2\,Rx - 2\,Tx)}{R2^2\,RY2} - \frac{(Ry - Ty)\,(Rz - Tz)^2\,(Rx - Tx)\,(2\,Rx - 2\,Tx)}{R2\,RY2^2} + \frac{(Ry - Ty)\,(Rz - Tz)^2}{R2\,RY2} \qquad \textbf{(4.3.23)}$$

$Z21_y := (Rx, Ry, Rz, Tx, Ty, Tz) \rightarrow diff\left(Z_2(Rx, Ry, Rz, Tx, Ty, Tz) \cdot Z_1(Rx, Ry, Rz, Tx, Ty, Tz), Ry\right)$:
$Z21_y(Rx, Ry, Rz, Tx, Ty, Tz)$;

$$\left((Rz - Tz)^2\,(Rx - Tx)\right) \Big/ \left((Rx^2 - 2\,Rx\,Tx + Tx^2 + Ry^2 - 2\,Ry\,Ty + Ty^2 + Rz^2 - 2\,Rz\,Tz + Tz^2)\,(Rx^2 - 2\,Rx\,Tx + Tx^2 + Ry^2 - 2\,Ry\,Ty + Ty^2)\right) - \left((Ry - Ty)\,(Rz - Tz)^2\,(Rx - Tx)\,(2\,Ry - 2\,Ty)\right) \Big/ \left((Rx^2 - 2\,Rx\,Tx + Tx^2 + Ry^2 - 2\,Ry\,Ty + Ty^2 + Rz^2 - 2\,Rz\,Tz + Tz^2)^2\,(Rx^2 - 2\,Rx\,Tx + Tx^2 + Ry^2 - 2\,Ry\,Ty + Ty^2)\right) - \left((Ry - Ty)\,(Rz - Tz)^2\,(Rx - Tx)\,(2\,Ry - 2\,Ty)\right) \Big/ \left((Rx^2 - 2\,Rx\,Tx + Tx^2 + Ry^2 - 2\,Ry\,Ty + Ty^2 + Rz^2 - 2\,Rz\,Tz + Tz^2)\,(Rx^2 - 2\,Rx\,Tx + Tx^2 + Ry^2 - 2\,Ry\,Ty + Ty^2)^2\right) \qquad \textbf{(4.3.24)}$$

(4.3.25)

$subs(Rx^2 - 2\,Rx\,Tx + Tx^2 + Ry^2 - 2\,Ry\,Ty + Ty^2 + Rz^2 - 2\,Rz\,Tz + Tz^2 = R2, \%)$:

$subs\left(Rx^2 - 2\,Rx\,Tx + Tx^2 + Ry^2 - 2\,Ry\,Ty + Ty^2 = RY2, \%\right):$
$subs\left((Rx - Tx)^2 + (Ry - Ty)^2 = RY2, \%\right);$

$$\frac{(Rz - Tz)^2\,(Rx - Tx)}{R2\,RY2} - \frac{(Ry - Ty)\,(Rz - Tz)^2\,(Rx - Tx)\,(2\,Ry - 2\,Ty)}{R2^2\,RY2} - \frac{(Ry - Ty)\,(Rz - Tz)^2\,(Rx - Tx)\,(2\,Ry - 2\,Ty)}{R2\,RY2^2} \quad \textbf{(4.3.26)}$$

$Z21_z := (Rx, Ry, Rz, Tx, Ty, Tz) \rightarrow diff\left(Z_2(Rx, Ry, Rz, Tx, Ty, Tz)\cdot Z_1(Rx, Ry, Rz, Tx, Ty, Tz), Rz\right):$
$Z21_z(Rx, Ry, Rz, Tx, Ty, Tz);$

$$(2\,(Ry - Ty)\,(Rz - Tz)\,(Rx - Tx))\Big/\left(\left(Rx^2 - 2\,Rx\,Tx + Tx^2 + Ry^2 - 2\,Ry\,Ty + Ty^2 + Rz^2 - 2\,Rz\,Tz + Tz^2\right)\left(Rx^2 - 2\,Rx\,Tx + Tx^2 + Ry^2 - 2\,Ry\,Ty + Ty^2\right)\right) - \left((Ry - Ty)\,(Rz - Tz)^2\,(Rx - Tx)\,(2\,Rz - 2\,Tz)\right)\Big/\left(\left(Rx^2 - 2\,Rx\,Tx + Tx^2 + Ry^2 - 2\,Ry\,Ty + Ty^2 + Rz^2 - 2\,Rz\,Tz + Tz^2\right)^2\left(Rx^2 - 2\,Rx\,Tx + Tx^2 + Ry^2 - 2\,Ry\,Ty + Ty^2\right)\right) \quad \textbf{(4.3.27)}$$

(4.3.28)

$subs\left(Rx^2 - 2\,Rx\,Tx + Tx^2 + Ry^2 - 2\,Ry\,Ty + Ty^2 + Rz^2 - 2\,Rz\,Tz + Tz^2 = R2, \%\right):$
$subs\left(Rx^2 - 2\,Rx\,Tx + Tx^2 + Ry^2 - 2\,Ry\,Ty + Ty^2 = RY2, \%\right):$
$subs\left((Rx - Tx)^2 + (Ry - Ty)^2 = RY2, \%\right);$

$$\frac{2\,(Ry - Ty)\,(Rz - Tz)\,(Rx - Tx)}{R2\,RY2} - \frac{(Ry - Ty)\,(Rz - Tz)^2\,(Rx - Tx)\,(2\,Rz - 2\,Tz)}{R2^2\,RY2} \quad \textbf{(4.3.29)}$$

z[3]*z[1] = -x[3]*x[1]

$Z31_x := (Rx, Ry, Rz, Tx, Ty, Tz) \rightarrow diff\left(Z_3(Rx, Ry, Rz, Tx, Ty, Tz)\cdot Z_1(Rx, Ry, Rz, Tx, Ty, Tz), Rx\right):$
$Z31_x(Rx, Ry, Rz, Tx, Ty, Tz);$

$$-((2\,Rx - 2\,Tx)\,(Rx - Tx)\,(Rz - Tz))\Big/\left(\left(Rx^2 - 2\,Rx\,Tx + Tx^2 + Ry^2 - 2\,Ry\,Ty + Ty^2 + Rz^2 - 2\,Rz\,Tz + Tz^2\right)\left(Rx^2 - 2\,Rx\,Tx + Tx^2 + Ry^2 - 2\,Ry\,Ty + Ty^2\right)\right) + \left(\left((Rx - Tx)^2 + (Ry - Ty)^2\right)(Rx - Tx)\,(Rz - Tz)\,(2\,Rx - 2\,Tx)\right)\Big/\left(\left(Rx^2 - 2\,Rx\,Tx + Tx^2 + Ry^2 \ldots + Ty^2\right)\right) + \left(\left((Rx - Tx)^2 + (Ry - Ty)^2\right)(Rx - Tx)\,(Rz - Tz)\,(2\,Rx - 2\,Tx)\right)\Big/\left(\left(Rx^2 - 2\,Rx\,Tx + Tx^2 + Ry^2 - 2\,Ry\,Ty + Ty^2 + Rz^2 - 2\,Rz\,Tz + Tz^2\right)\left(Rx^2 - 2\,Rx\,Tx + Tx^2 + Ry^2 - 2\,Ry\,Ty + Ty^2\right)^2\right) - \left(\left((Rx - Tx)^2 + (Ry - Ty)^2\right)(Rz - Tz)\right) / \ldots - 2\,Ry\,Ty + Ty^2))$$

(4.3.31)

$subs\left(Rx^2 - 2\,Rx\,Tx + Tx^2 + Ry^2 - 2\,Ry\,Ty + Ty^2 + Rz^2 - 2\,Rz\,Tz + Tz^2 = R2, \%\right):$
$subs\left(Rx^2 - 2\,Rx\,Tx + Tx^2 + Ry^2 - 2\,Ry\,Ty + Ty^2 = RY2, \%\right):$
$subs\left((Rx - Tx)^2 + (Ry - Ty)^2 = RY2, \%\right);$

$$\frac{(Rx-Tx)\,(Rz-Tz)\,(2\,Rx-2\,Tx)}{R2^2}-\frac{Rz-Tz}{R2} \quad \textbf{(4.3.32)}$$

$Z31_y := (Rx, Ry, Rz, Tx, Ty, Tz) \rightarrow diff\big(Z_3(Rx, Ry, Rz, Tx, Ty, Tz)\cdot Z_1(Rx, Ry, Rz, Tx, Ty, Tz), Ry\big)$:

$Z31_y(Rx, Ry, Rz, Tx, Ty, Tz)$;

$$-\big((2\,Ry-2\,Ty)\,(Rx-Tx)\,(Rz-Tz)\big)\Big/\big((Rx^2-2\,Rx\,Tx+Tx^2+Ry^2-2\,Ry\,Ty+Ty^2+Rz^2-2\,Rz\,Tz+Tz^2)\,(Rx^2-2\,Rx\,Tx+Tx^2+Ry^2-2\,Ry\,Ty+Ty^2)\big)+\big(\big((Rx-Tx)^2+(Ry-Ty)^2\big)\,(Rx-Tx)\,(Rz-Tz)\,(2\,Ry-2\,Ty)\big)\Big/\big((Rx^2-2\,Rx\,Tx+Tx^2+Ry^2+Ty^2)\big)+\big(\big((Rx-Tx)^2+(Ry-Ty)^2\big)\,(Rx-Tx)\,(Rz-Tz)\,(2\,Ry-2\,Ty)\big)\Big/\big((Rx^2-2\,Rx\,Tx+Tx^2+Ry^2-2\,Ry\,Ty+Ty^2+Rz^2-2\,Rz\,Tz+Tz^2)\,(Rx^2-2\,Rx\,Tx+Tx^2+Ry^2-2\,Ry\,Ty+Ty^2)^2\big) \quad \textbf{(4.3.34)}$$

$subs\big(Rx^2-2\,Rx\,Tx+Tx^2+Ry^2-2\,Ry\,Ty+Ty^2+Rz^2-2\,Rz\,Tz+Tz^2=R2, \%\big)$:
$subs\big(Rx^2-2\,Rx\,Tx+Tx^2+Ry^2-2\,Ry\,Ty+Ty^2=RY2, \%\big)$:
$subs\big((Rx-Tx)^2+(Ry-Ty)^2=RY2, \%\big)$;

$$\frac{(Rx-Tx)\,(Rz-Tz)\,(2\,Ry-2\,Ty)}{R2^2} \quad \textbf{(4.3.35)}$$

$Z31_z := (Rx, Ry, Rz, Tx, Ty, Tz) \rightarrow diff\big(Z_3(Rx, Ry, Rz, Tx, Ty, Tz)\cdot Z_1(Rx, Ry, Rz, Tx, Ty, Tz), Rz\big)$:

$Z31_z(Rx, Ry, Rz, Tx, Ty, Tz)$;

$$\big(\big((Rx-Tx)^2+(Ry-Ty)^2\big)\,(Rx-Tx)\,(Rz-Tz)\,(2\,Rz-2\,Tz)\big)\Big/\big((Rx^2-2\,Rx\,Tx+Tx^2+Ry^2-2\,Ry\,Ty+Ty^2+Rz^2-2\,Rz\,Tz+Tz^2)^2\,(Rx^2-2\,Rx\,Tx+Tx^2+Ry^2-2\,Ry\,Ty+Ty^2)\big)-\big(\big((Rx-Tx)^2+(Ry-Ty)^2\big)\,(Rx-Tx)\big)\Big/\big((Rx^2-2\,Rx\,Tx+Tx^2+Ry^2-2\,Ry\,Ty+Ty^2+Rz^2-2\,Rz\,Tz+Tz^2)\,(Rx^2-2\,Rx\,Tx+Tx^2+Ry^2-2\,Ry\,Ty+Ty^2)\big) \quad \textbf{(4.3.36)}$$

(4.3.37)

$subs\big(Rx^2-2\,Rx\,Tx+Tx^2+Ry^2-2\,Ry\,Ty+Ty^2+Rz^2-2\,Rz\,Tz+Tz^2=R2, \%\big)$:
$subs\big(Rx^2-2\,Rx\,Tx+Tx^2+Ry^2-2\,Ry\,Ty+Ty^2=RY2, \%\big)$:
$subs\big((Rx-Tx)^2+(Ry-Ty)^2=RY2, \%\big)$;

$$\frac{(Rx-Tx)\,(Rz-Tz)\,(2\,Rz-2\,Tz)}{R2^2}-\frac{Rx-Tx}{R2} \quad \textbf{(4.3.38)}$$

z[3]*z[2] = -x[3]*x[2]

$Z32_x := (Rx, Ry, Rz, Tx, Ty, Tz) \rightarrow diff\big(Z_3(Rx, Ry, Rz, Tx, Ty, Tz)\cdot Z_2(Rx, Ry, Rz, Tx, Ty, Tz), Rx\big)$:

$Z32_x(Rx, Ry, Rz, Tx, Ty, Tz)$;

$$-\big((2\,Rx-2\,Tx)\,(Ry-Ty)\,(Rz-Tz)\big)\Big/\big((Rx^2-2\,Rx\,Tx+Tx^2+Ry^2-2\,Ry\,Ty+Ty^2+Rz^2-2\,Rz\,Tz+Tz^2)\,(Rx^2-2\,Rx\,Tx+Tx^2+Ry^2-2\,Ry\,Ty+Ty^2)\big)$$

$$+\left(\left((Rx-Tx)^2+(Ry-Ty)^2\right)(Ry-Ty)(Rz-Tz)(2\,Rx-2\,Tx)\right)\Big/\left((Rx^2-2\,Rx\,Tx+Tx^2+Ry^2 + Ty^2)\right)+\left(\left((Rx-Tx)^2+(Ry-Ty)^2\right)(Ry-Ty)(Rz-Tz)(2\,Rx -2\,Tx)\right)\Big/\left((Rx^2-2\,Rx\,Tx+Tx^2+Ry^2-2\,Ry\,Ty+Ty^2+Rz^2-2\,Rz\,Tz +Tz^2)(Rx^2-2\,Rx\,Tx+Tx^2+Ry^2-2\,Ry\,Ty+Ty^2)^2\right)$$

(4.3.40)

$subs\left(Rx^2-2\,Rx\,Tx+Tx^2+Ry^2-2\,Ry\,Ty+Ty^2+Rz^2-2\,Rz\,Tz+Tz^2=R2,\%\right):$
$subs\left(Rx^2-2\,Rx\,Tx+Tx^2+Ry^2-2\,Ry\,Ty+Ty^2=RY2,\%\right):$
$subs\left((Rx-Tx)^2+(Ry-Ty)^2=RY2,\%\right);$

$$\frac{(Ry-Ty)(Rz-Tz)(2\,Rx-2\,Tx)}{R2^2} \quad \textbf{(4.3.41)}$$

$Z32_y := (Rx, Ry, Rz, Tx, Ty, Tz) \rightarrow diff\left(Z_3(Rx, Ry, Rz, Tx, Ty, Tz)\cdot Z_2(Rx, Ry, Rz, Tx, Ty, Tz), Ry\right):$
$Z32_y(Rx, Ry, Rz, Tx, Ty, Tz);$

$$-\left((2\,Ry-2\,Ty)(Ry-Ty)(Rz-Tz)\right)\Big/\left((Rx^2-2\,Rx\,Tx+Tx^2+Ry^2-2\,Ry\,Ty+Ty^2 +Rz^2-2\,Rz\,Tz+Tz^2)(Rx^2-2\,Rx\,Tx+Tx^2+Ry^2-2\,Ry\,Ty+Ty^2)\right) +\left(\left((Rx-Tx)^2+(Ry-Ty)^2\right)(Ry-Ty)(Rz-Tz)(2\,Ry-2\,Ty)\right)\Big/\left((Rx^2-2\,Rx\,Tx+Tx^2+Ry^2 + Ty^2)\right)+\left(\left((Rx-Tx)^2+(Ry-Ty)^2\right)(Ry-Ty)(Rz-Tz)(2\,Ry -2\,Ty)\right)\Big/\left((Rx^2-2\,Rx\,Tx+Tx^2+Ry^2-2\,Ry\,Ty+Ty^2+Rz^2-2\,Rz\,Tz +Tz^2)(Rx^2-2\,Rx\,Tx+Tx^2+Ry^2-2\,Ry\,Ty+Ty^2)^2\right)-\left(\left((Rx-Tx)^2+(Ry-Ty)^2\right)(Rz-Tz)\right)/ -2\,Ry\,Ty+Ty^2)\big)$$

(4.3.43)

$subs\left(Rx^2-2\,Rx\,Tx+Tx^2+Ry^2-2\,Ry\,Ty+Ty^2+Rz^2-2\,Rz\,Tz+Tz^2=R2,\%\right):$
$subs\left(Rx^2-2\,Rx\,Tx+Tx^2+Ry^2-2\,Ry\,Ty+Ty^2=RY2,\%\right):$
$subs\left((Rx-Tx)^2+(Ry-Ty)^2=RY2,\%\right);$

$$\frac{(Ry-Ty)(Rz-Tz)(2\,Ry-2\,Ty)}{R2^2}-\frac{Rz-Tz}{R2} \quad \textbf{(4.3.44)}$$

$Z32_z := (Rx, Ry, Rz, Tx, Ty, Tz) \rightarrow diff\left(Z_3(Rx, Ry, Rz, Tx, Ty, Tz)\cdot Z_2(Rx, Ry, Rz, Tx, Ty, Tz), Rz\right):$
$Z32_z(Rx, Ry, Rz, Tx, Ty, Tz);$

$$\left(\left((Rx-Tx)^2+(Ry-Ty)^2\right)(Ry-Ty)(Rz-Tz)(2\,Rz-2\,Tz)\right)\Big/\left((Rx^2-2\,Rx\,Tx +Tx^2+Ry^2-2\,Ry\,Ty+Ty^2+Rz^2-2\,Rz\,Tz+Tz^2)^2(Rx^2-2\,Rx\,Tx+Tx^2+Ry^2 -2\,Ry\,Ty+Ty^2)\right)-\left(\left((Rx-Tx)^2+(Ry-Ty)^2\right)(Ry-Ty)\right)\Big/\left((Rx^2 -2\,Rx\,Tx+Tx^2+Ry^2-2\,Ry\,Ty+Ty^2+Rz^2-2\,Rz\,Tz+Tz^2)(Rx^2-2\,Rx\,Tx +Tx^2+Ry^2-2\,Ry\,Ty+Ty^2)\right) \quad \textbf{(4.3.45)}$$

(4.3.46)

$subs(Rx^2 - 2\,Rx\,Tx + Tx^2 + Ry^2 - 2\,Ry\,Ty + Ty^2 + Rz^2 - 2\,Rz\,Tz + Tz^2 = R2, \%)$:
$subs(Rx^2 - 2\,Rx\,Tx + Tx^2 + Ry^2 - 2\,Ry\,Ty + Ty^2 = RY2, \%)$:
$subs((Rx - Tx)^2 + (Ry - Ty)^2 = RY2, \%)$;

$$\frac{(Ry - Ty)\,(Rz - Tz)\,(2\,Rz - 2\,Tz)}{R2^2} - \frac{Ry - Ty}{R2} \tag{4.3.47}$$
