# Supplementary figures and images for: Interface of the Polarizable Continuum Model of Solvation with Semi-Empirical Methods in the GAMESS Program
Source: PLoS One. 2013 Jul 2;8(7):e67725. doi: 10.1371/journal.pone.0067725 (PMC3699639; doi:10.1371/journal.pone.0067725)

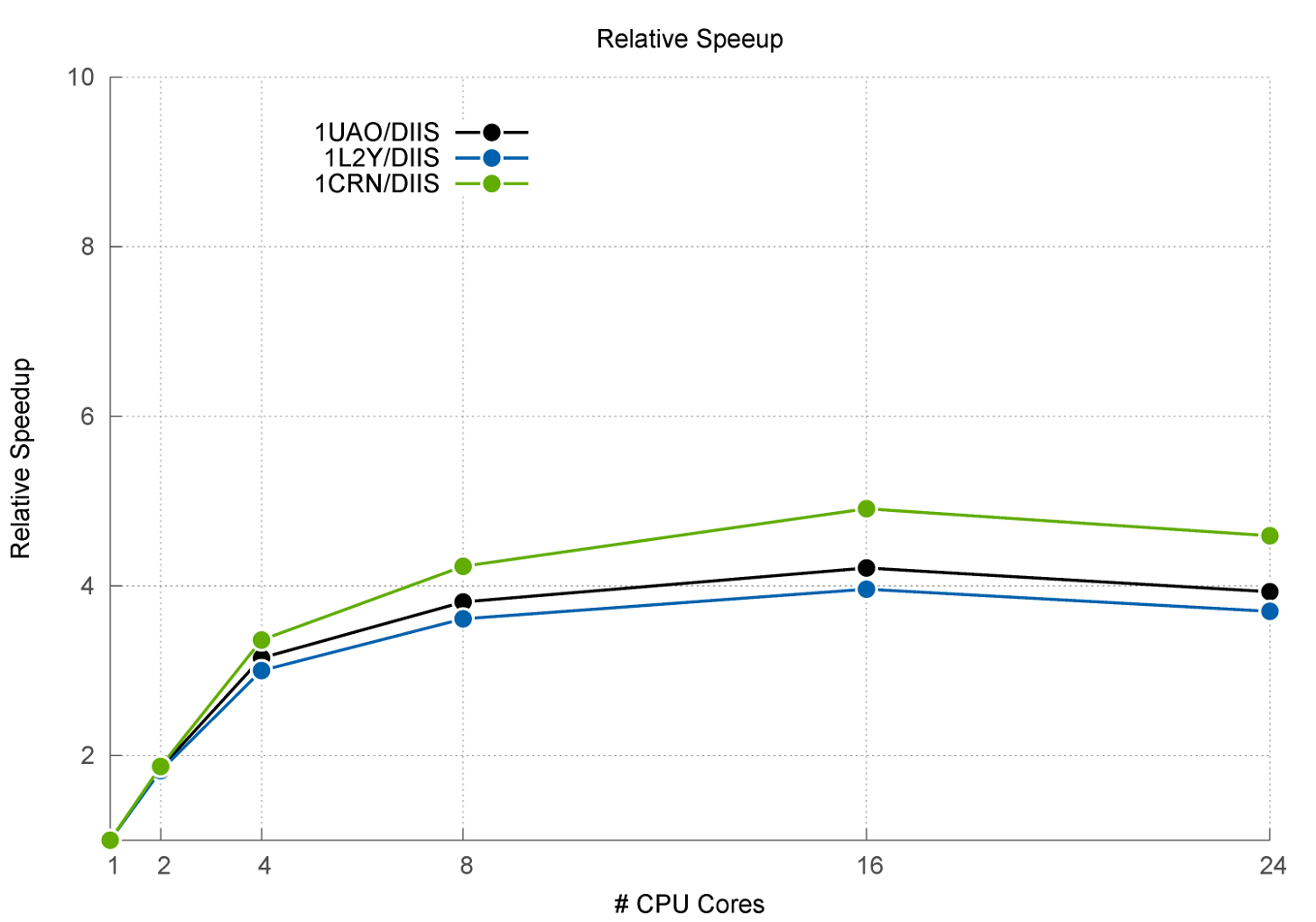

Supplement: Figure S1 — Speedup by using multiple cores single gas phase gradient evaluation. (TIFF) [file pone.0067725.s002.tiff]
